# Tectonic evolution of the Nootka fault zone and deformation of the shallow subducted Explorer plate in northern Cascadia as revealed by earthquake distributions and seismic tomography

**DOI:** 10.1038/s41598-023-33310-z

**Published:** 2023-05-15

**Authors:** Jesse Hutchinson, Honn Kao, Michael Riedel, Koichiro Obana, Kelin Wang, Shuichi Kodaira, Tsutomu Takahashi, Yojiro Yamamoto

**Affiliations:** 1 School of Earth and Ocean Sciences, University of Victoria, Victoria, BC V8P 5C2 Canada; 2 Pacific Geoscience Centre, Geological Survey of Canada, Natural Resources Canada, Sidney, BC V8L 4B2 Canada; 3 GEOMAR Helmholtz-Centre for Ocean Research Kiel, Kiel, Germany; 4 Japan Agency for Marine-Earth Science and Technology (JAMSTEC), Yokohama, Japan; 5 Present Address: Ocean Networks Canada, University of Victoria, Victoria, BC V8P 5C2 Canada

**Keywords:** Tectonics, Seismology

## Abstract

At the northern Cascadia subduction zone, the subducting Explorer and Juan de Fuca plates interact across a transform deformation zone, known as the Nootka fault zone (NFZ). This study continues the Seafloor Earthquake Array Japan Canada Cascadia Experiment to a second phase (SeaJade II) consisting of nine months of recording of earthquakes using ocean-bottom and land-based seismometers. In addition to mapping the distribution of seismicity, including an M_W_ 6.4 earthquake and aftershocks along the previously unknown Nootka Sequence Fault, we also conducted seismic tomography, which delineates the geometry of the shallow subducting Explorer plate (ExP). We derived hundreds of high-quality focal mechanism solutions from the SeaJade II data. The mechanisms manifest a complex regional tectonic state, with normal faulting of the ExP west of the NFZ, left-lateral strike-slip behaviour of the NFZ, and reverse faulting within the overriding plate above the subducting Juan de Fuca plate. Using data from the combined SeaJade I and II catalogs, we have performed double-difference hypocentre relocations and found seismicity lineations to the southeast of, and oriented 18° clockwise from, the subducted NFZ, which we interpret to represent less active small faults off the primary faults of the NFZ. These lineations are not optimally oriented for shear failure in the regional stress field, which we inferred from averaged focal mechanism solutions, and may represent paleo-configurations of the NFZ. Further, active faults interpreted from seismicity lineations within the subducted plate, including the Nootka Sequence Fault, may have originated as conjugate faults within the paleo-NFZ.

## Introduction

The Cascadia subduction zone (CSZ) is defined by the subduction of the Explorer (ExP), Juan de Fuca (JdF), and Gorda plates, formerly parts of the Farallon plate, beneath the western margin of the North America plate (NAm) from northern Vancouver Island to northern California^1^. The CSZ is capable of producing M_W_ 9+ earthquakes, the last of which occurred during 1700 AD^2,3^, but it is unusually quiescent at present. Recent research that involved the use of ocean-bottom seismometers (OBS)^4,5^ has focused primarily on studying the JdF. Few OBS experiments, however, have focused on the ExP.

**Figure 1:**
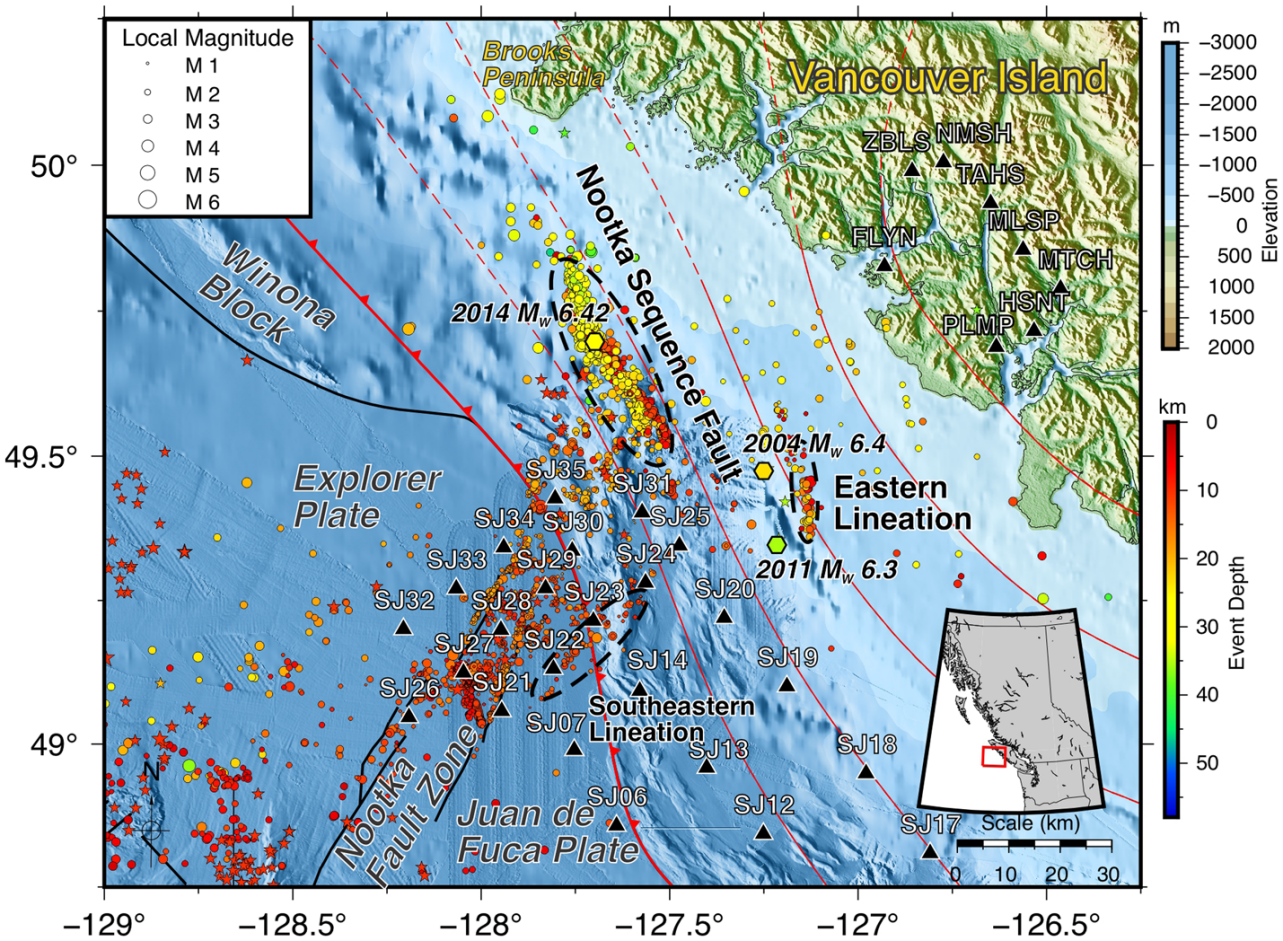
Map of the study area of SeaJade II and the Nootka fault zone, generated with GMT^37^. The inset shows regional geography with the map area outlined using a red box. In this figure and all map view figures in this paper, earthquakes relocated with the double-difference method^11,12^ from this study are shown as circles, with colour indicating depth. Historical earthquakes with magnitudes >  = 4 are shown as colour-filled stars (Earthquakes Canada). Historic large magnitude earthquakes from 2004, 2011, and 2014 are labelled and shown as hexagons. Shape sizes are indicative of earthquake magnitude. Stations from both the ocean-bottom and land components of SeaJade II are shown as black triangles. The subduction front of the Cascadia subduction zone is indicated by the curving red line running from northwest to southeast with triangular teeth pointing in the direction of subduction^42^. The location of the Winona block is from Gao et al.^43^ and Sypus^44^. The thinner red lines are 5 km contours of the plate interface based on the interpretation of Audet et al.^45^, with dashed contours representing highly uncertain interface depths. Several areas important to discussion are circled with dashed black lines and labelled.

The Seafloor Earthquake Array Japan Canada Cascadia Experiment (SeaJade) is designed to monitor the shallow Cascadia subduction interface and the unsubducted and subducted parts of the Juan de Fuca and Explorer plates, which are separated by the Nootka fault zone (NFZ) (Fig. 1). Up to thirty-five OBS were deployed off the west coast of Vancouver Island, Canada in two phases in 2010 and 2014.

Data from the first deployment (SeaJade I), during July–September 2010, allowed for the delineation of the NFZ through hypocentre distributions. The NFZ is bounded to the north and south by two mature primary faults that extend through Moho and run NE-SW, between which lie several less developed secondary conjugate faults. SeaJade I also provided insight into the depths to interfaces across which there is a contrast in velocity including the oceanic Moho within the subducting plate determined via reflected PS and SP phases. It was found that the oceanic crust is approximately 7 km thick just seaward of the subduction front^6^.

The second deployment (SeaJade II) recorded data for nine months, beginning in January 2014. Several OBSs were placed further north of the NFZ than during SeaJade I. During the same time, land seismometers were deployed in the Nootka sound region along the west coast of Vancouver Island to accompany the OBS network (Fig. 1). Locations of the seismometers are included in the Supplemental Table S1.

One of the most important objectives of SeaJade II was to define the shallow geometry of the subducted JdF and ExP. Our previous analysis of the SeaJade II data focus primarily on an M_W_ 6.4 earthquake that occurred on 24 April 2014, during our recording period, and the associated aftershocks referred to as the Nootka Sequence^7^. From these results, we delineated a fault within the subducted ExP. The hypocentre distribution of the Nootka Sequence has revealed that the subducting plate is bending downward to the northwest, nearly perpendicular to the direction of subduction. The results show that the NFZ not only marks a change in subduction rate and direction, but it is also a transition from the more shallowly dipping subduction to the south, to the more steeply dipping subduction to the north. Substantial evidence is provided for this change in plate geometry in the analysis of the hypocentre distribution and in the complexity of focal mechanisms^7^.

In this study, we further constrain the northwest dip of the ExP with the addition of three-dimensional (3-D) seismic tomography and relocation of the earthquakes within this 3D velocity model. The new seismic tomography combines the complete data from both SeaJade I and II to maximize resolution and spatial coverage. The tomography maps regions of low velocities that closely follow the hypocentre distribution of the Nootka Sequence, clearly distinguishing the two downgoing oceanic plates. Merrill et al.^8^ also performed a seismic tomography inversion for their study of northern Cascadia, however, their results have a lower resolution and focus more broadly across the subduction front, and they did not image the northward bending ExP, as we do in this study.

Additionally, the detailed analysis of the combined data highlights hypocentre lineations previously unnoticed within the NFZ. These seismogenic structures may reflect the tectonic characteristics of the paleo-NFZ when shear deformation was more broadly distributed and did not lead to the consolidation and development of deeper mature faults. From this distribution and the orientation of focal mechanism trends, we can infer the developmental history of the NFZ.

## Methods and data analysis

We include the hypocentre distributions and focal mechanism solutions reported in our previous two studies^6,7^. For this study, we expand the number of relocated hypocentres from 2,609 to 3,918 by adding events from before and after the Nootka Sequence and increasing the number of high-quality focal mechanism solutions to 1,089 from 502 during the Nootka Sequence. The associated arrival and relocated event data are provided in Supplemental Tables A1 and A2, and the focal mechanism data are provided in Supplemental Table B1. We compute initial locations using the 1-D velocity model from Spence et al.^9^ and the GENLOC location package^10^ from the Antelope software suite. For further details, our initial location procedure is documented in Hutchinson et al.^7^. For the complete SeaJade II dataset, we calculate location uncertainty by bootstrapping 80% of the associated phases over 1000 iterations for each event. Upon the removal of outliers, we found the average 3-sigma values for the major and minor error ellipses to be 2.9 and 1.2 km, respectively, and the depth error to be 4.5 km.

We utilize the TomoDD method to better determine the distribution of hypocentres and 3-D seismic velocity model for our study area^11^. TomoDD jointly determines double-difference hypocentre solutions and inverts for the 3-D distribution of seismic velocities. The double-difference relocations are determined by utilizing travel-time differences of seismic phases (*P* and/or *S*) and waveform cross-correlations^12^. These data allow for the relative relocation of events to one another. TomoDD also accounts for the absolute locations of hypocentres so that both the accuracy and precision of hypocentre locations are greatly improved.

We determine the 3-D velocity model for a 200-by-200 km area with 2.1 km spacing centred around 49.25° N, 127.75° W. The vertical nodes are spaced more closely, at 1 km intervals from the depths of 0 km to 52 km below sea level with the exception of the shallowest node, which is placed 1.3 km above sea level to provide ample boundary padding. Any events relocated above the seafloor are rejected by the procedure. To test for the sensitivity of the velocity model to node spacing, we also perform joint inversions with horizontal node spacings of 3, 4, or 5 km. We find that large low- and high-velocity structures are comparable between the different spacings, however the detailed tomography suffers at larger node intervals with a loss of resolution.

We incorporate event data from the SeaJade I^6^ and II (from this and our previous study^7^) catalogues for a more robust seismic tomography model. The combined 4573 events were input with ~ 136,000 associated *P-* and *S*-phases. Typical seismic waveforms and phase picks are shown in Figure S1. Of these events, 3918 could be relocated. Double-difference relocations and joint determination of 3-D velocities and relocations were alternated between steps of iterations. In total, we perform 78 iterations to calculate the final 3-D *P*- and *S*-velocity models. Parameters used for these computations are provided in Supplement C, while the V_P_ and V_S_ data are provided in Supplemental Tables C1 and C2, respectively. We also calculate V_P_/V_S_ ratios for each grid cell from the *P*- and *S*-velocities. We compute a mean V_P_/V_S_ ratio of 1.73 ± 0.03. Because we observed nearly equal numbers of *P*- and *S*-phases, and both types of phases were well-picked (see Figure S1), we decide that this is the best method for determining the V_P_/V_S_ ratio, unlike cases with poorer *S*-wave datasets^11,13,14^. It should be noted that this does not completely eliminate a potential bias due to different ray paths and volumes sampled by both wave types.

We quantitatively compare the location errors before and after applying TomoDD. We estimate hypocentral location errors before TomoDD by the bootstrapping method described above. With TomoDD, errors are most accurately determined by examining subsets of the entire dataset with singular-value decomposition (SVD, 3-sigma values). We select two representative subsets of events, based on different OBS coverage. The northern subset of 116 events is taken from the northwestern segment of the Nootka Sequence area described in Hutchinson et al.^7^. For this subset, the bootstrapping-determined 3-sigma values for the major, minor, and depth of the error ellipsoids are, on average, 3.17, 1.28 and 7.77 km, respectively. By comparison, the average errors for the X, Y, and depth axes of the error ellipsoids after TomoDD are 0.66, 0.67, and 1.22 km, respectively.

The southern subset consists of 120 events from the southeastern segment of the Nootka Sequence area where the OBS coverage is better. For this subset, we find significantly lower errors with average errors for the X, Y, and depth axes of the error ellipses of 0.10, 0.14, and 0.15 km, respectively. By comparison, the bootstrapping errors have average 3-sigma values for the major, minor, and depth of the error ellipses of 2.92, 1.22 and 3.41 km, respectively. Overall, TomoDD relocations have reduced the location error by up to an order of magnitude.

We derive focal mechanism solutions for the entire SeaJade II catalog (including those from Hutchinson et al.^7^) using the program HASH^15,16^. We use relocated hypocentres and velocity dependent raypaths to calculate the azimuth and take-off angle for our calculations. To ensure high-quality results, we use the peak displacements of *P*- and *S*-phases to calculate *S*/*P* amplitude ratios. Events with 8 or more clearly identifiable first-motion picks (a qualitative minimum requirement for HASH) are selected for calculating focal mechanism solutions. We found a total of 1448 solutions (Supplemental Table B1), of which 1089 are considered A-ranked, or the highest quality solutions on a scale of A to F, with F being the worst.

## Results and interpretations

### Hypocentre distribution

Relocated hypocentres are shown in Fig. 1. Seaward of the subduction front, most of the seismicity is concentrated within the NFZ, with some diffuse epicentres in the ExP and JdF. Landward of the subduction front, most seismicity is located within the Nootka Sequence, with another, smaller concentration of events east of the southeastern terminus of the Nootka Sequence.

**Figure 2:**
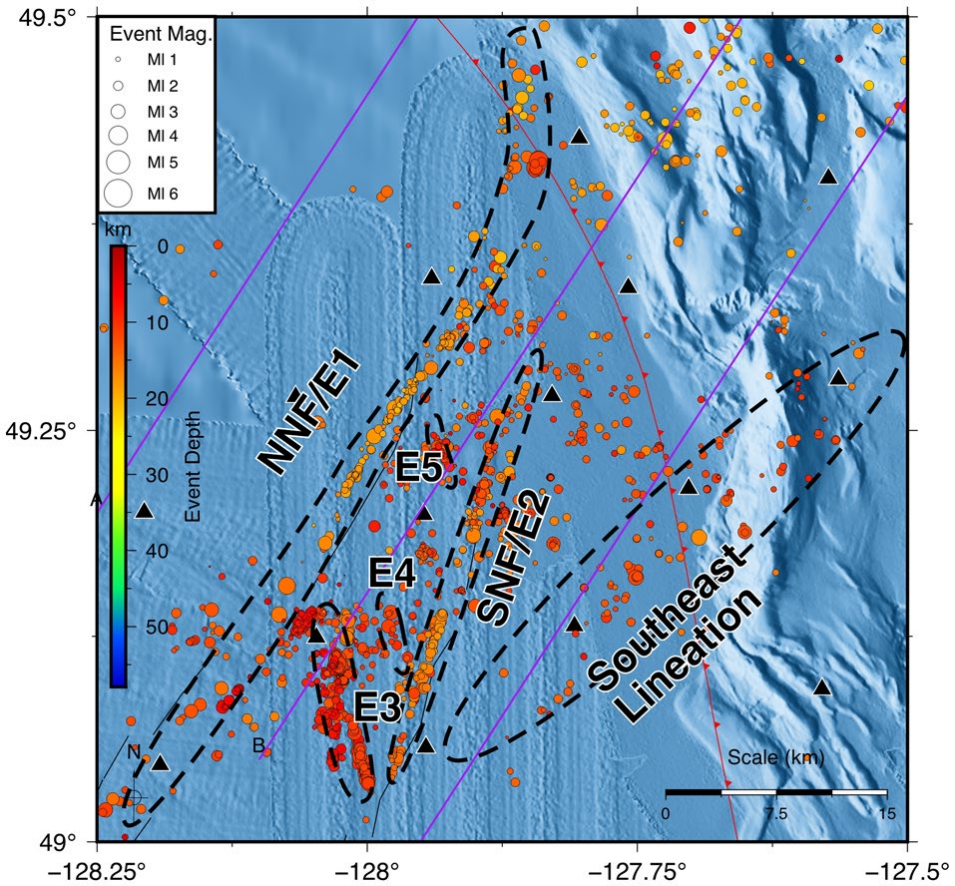
Detailed map of hypocentres focused on the Nootka fault zone, generated with GMT^37^. Purple lines are vertical transects shown in Fig. 5. Seismic features, including faults and more diffuse lineations, are outlined with black dashed lines and labelled after the conventions established in^6^. E1 and E2 are also labelled NNF and SNF, for northwestern and southeastern Nootka faults, after Rohr et al.^19^. See Fig. 1 for the explanation of other features on the map.

As inferred from SeaJade I observations^6^, the Nootka fault zone is comprised of many faults, identified as primary and secondary. The primary faults strike roughly NE-SW and extend the length of the NFZ. Secondary conjugate faults (labelled E3-E5 in Fig. 2) are located between the primary faults and strike roughly NW–SE, sub-perpendicular to the strike of the primary faults. These faults are represented by lineations of hypocentres, which can be seen in Figs. 1 and 2. Approximate fault strike, dip, width, and length for faults are provided in Table 1.

**Table 1:** Hypocentre lineations/zones and associated attributes.

Lineation/Zone	Strike	Dip	Lineation Length (km)	Lineation Width (km)	Number of Events	Earthquake Depth Range (km)	Earthquake Magnitude Range (M_L_)
E1 or NNF^a,b^	33.3° ± 0.4°	81.1° ± 2.8°	~ 30	0.9	~ 160	16–20	0.8–3.7
E1 NE	13.9° ± 1.7°		17	3	50	12–21	0.6–2.9
E2 or SNF^c^	198.8° ± 0.3°	81.2 ± 1.3	29	1.5	~ 190	11–19	0.6–3.2
E3	163.3° ± 0.1°	80.9° ± 1.4°	9.3	0.8	~ 450	9–12	0.6–4.5
E4	153.2° ± 1.4°	74.2° ± 2.6°	9.7	1.7	60	9–15	0.5–2.1
E5	152.1° ± 1.0°	82.8 ± 3.1	9.4	1.7	70	10.5–13.5	0.8–2.2
Eastern Lineation	170° ± 2.4°	84.7° ± 0.7°	17	3	~ 30	5–23	1.2–3.8
Southeastern Lineation	43.8° ± 1.2°	Too Diffuse	37	9	~ 110	8–16	0.7–3.4
ExP Strike-Slip	49.5° ± 1.9°	Too Diffuse	47	11	40	8–20	1.2–3.4
ExP	N/A	N/A	N/A	N/A	~ 80	10–22	0.8–3.4
JdF	N/A	N/A	N/A	N/A	~ 15	5–16	1.4–3.0
NW Nootka Sequence	157.7° ± 0.5°	Nearly Vertical	24.7	3–6.5	~ 610	9–17 (overriding), 20–36 (subducting)	1.6–6.4
SE Nootka Sequence	142.1° ± 0.4°	Nearly Vertical	29.3	3–6.5	~ 550	5–15 (overriding), 15–27 (subducting)	1.2–4.8

^a^Near to the subduction front, the NNF appears to change direction to a more northerly strike. Due to the small number of earthquakes, the strike has not been calculated.

^b^NNF is an abbreviation for the northwestern Nootka fault.

^c^SNF is an abbreviation for the southeastern Nootka fault.

Since SeaJade I, several previously undetected zones of seismicity have been observed. The Nootka Sequence fault, described in Hutchinson et al.^7^, is the most obvious of these features, extending over 55 km in length with a width in excess of 6 km.

Another seismicity lineation is labelled as the Eastern Lineation (Fig. 1) is located 25 km east of the southern Nootka Sequence fault, extending approximately 17 km in length along a more northerly trend in comparison to the Nootka Sequence fault. This distribution of seismicity ranges in depth from 5 to 24 km and coincides with several historical earthquakes, including M_W_ 6.4 and 6.3 oblique strike-slip events that occurred on 19 July 2004 and 9 September 2011, respectively (based on the National Earthquake Database compiled by Natural Resources Canada, http://earthquakescanada.nrcan.gc.ca, last accessed August 2021). The occurrence of such large events near the lineation implies that it is likely a fault that has consistently reactivated and can generate M_W_ 6+ earthquakes (Fig. 1).

Approximately 10–12 km southeast of the southern primary fault of the NFZ is a less well-lineated feature, labelled as the Southeastern Lineation. It extends for nearly 15 km before encountering the subduction front (Fig. 2). Earthquake hypocentres continue to follow the trend of this lineation northeast of the subduction front for at least another 20 km. Because these earthquakes are confined to smaller range of depths (extending to the depth of ~ 16 km) than those delineating the northern and southern primary faults of the NFZ (extending to the depth of 20 km), and are more broadly distributed, we consider the fault responsible for these events to be less mature.

### Seismic tomography

To test the resolution of our seismic tomography model, we have performed several checkerboard tests with cube sizes of 10, 8, 5, or 3 km. We calculate synthetic travel-times for each phase using the finite-difference scheme of Hole and Zelt^17^. Checkerboard squares are set to have alternating velocities of ± 5% of 5 km/s. We find that the minimum resolvable scale is generally 5 km, although 3 km cubes can be resolved in areas with the greatest raypath densities. The results from the 10, 8, 5, and 3 km resolution tests are shown in Figure S2a, b, c, and d, respectively. It should be noted that the checkerboard test results are limited due strictly to calculation only with synthetic travel-times. We do not synthesize waveform cross-correlation coefficients for the synthetic test, which our final tomography model utilizes for further precision in the double-differencing method. Therefore, it is likely that our model has better resolution than indicated by the checkerboard tests.

The derivative-weight sum (DWS) of the raypath density, as determined with TomoDD, also provides a method of determining the areas within the tomography model with the highest resolution. We calculate the base-10 logarithm for each DWS value and contour the results in Figure S2. We have found higher DWS values correlate to better-resolved areas in the checkerboard output and provide a valuable means for discussing areas with sufficient resolution.

**Figure 3:**
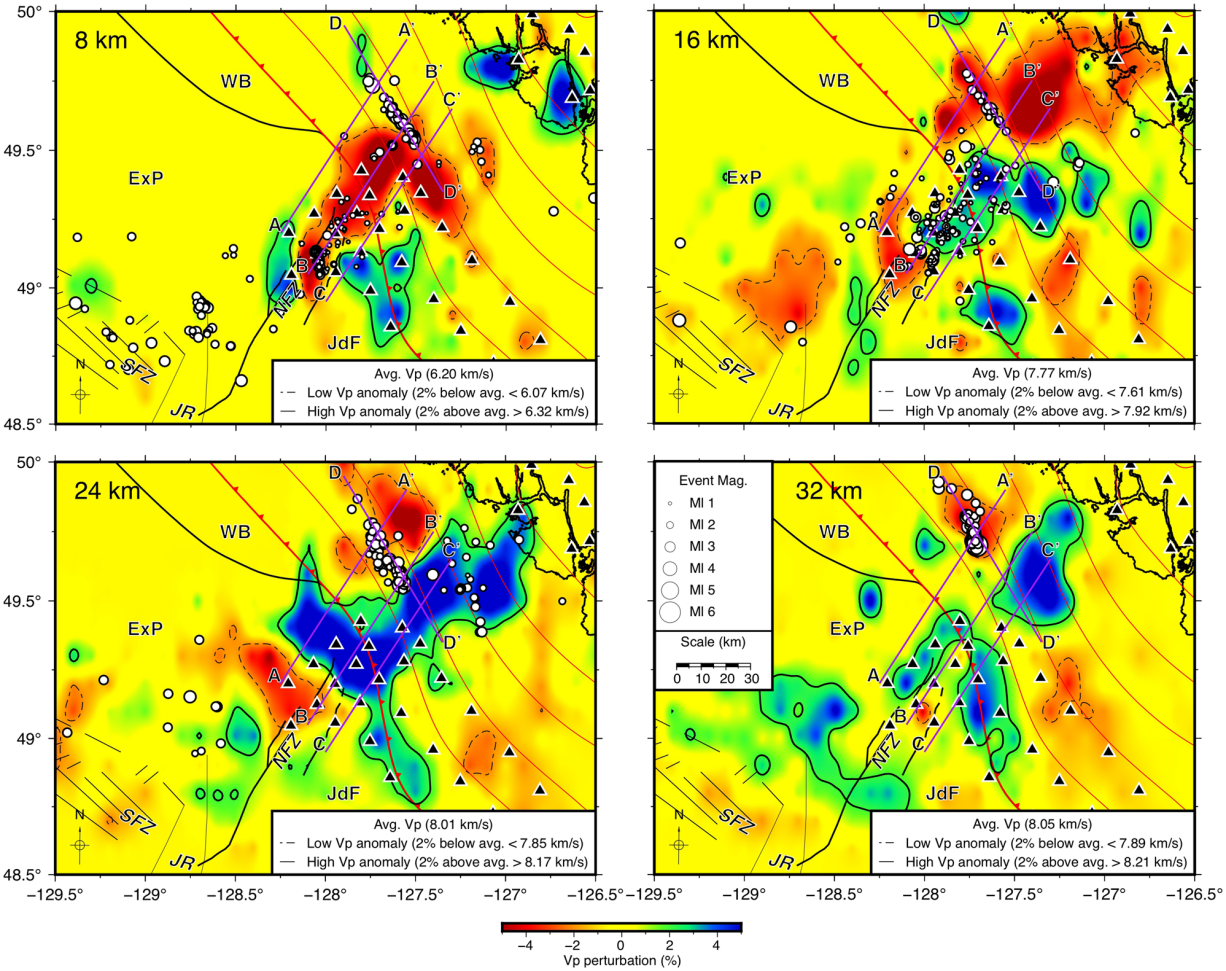
Depth slices of Vp of seismic tomography, generated with GMT^37^. From the upper left to the lower right, depths increase by 8 km intervals from 8 to 32 km. Hypocentres within a vertical distance of 1 km are shown as white-filled circles, with size indicating magnitude. Velocity anomalies are contoured where they are 2% above or below mean P-velocities for the depth slice. Low and high velocity anomalies are indicated by red and blue colours, as well as by dashed and solid contours, respectively. The purple lines show the locations of vertical cross-sections in Fig. 5. See Fig. 1 for the explanation of other features on the map.

**Figure 4:**
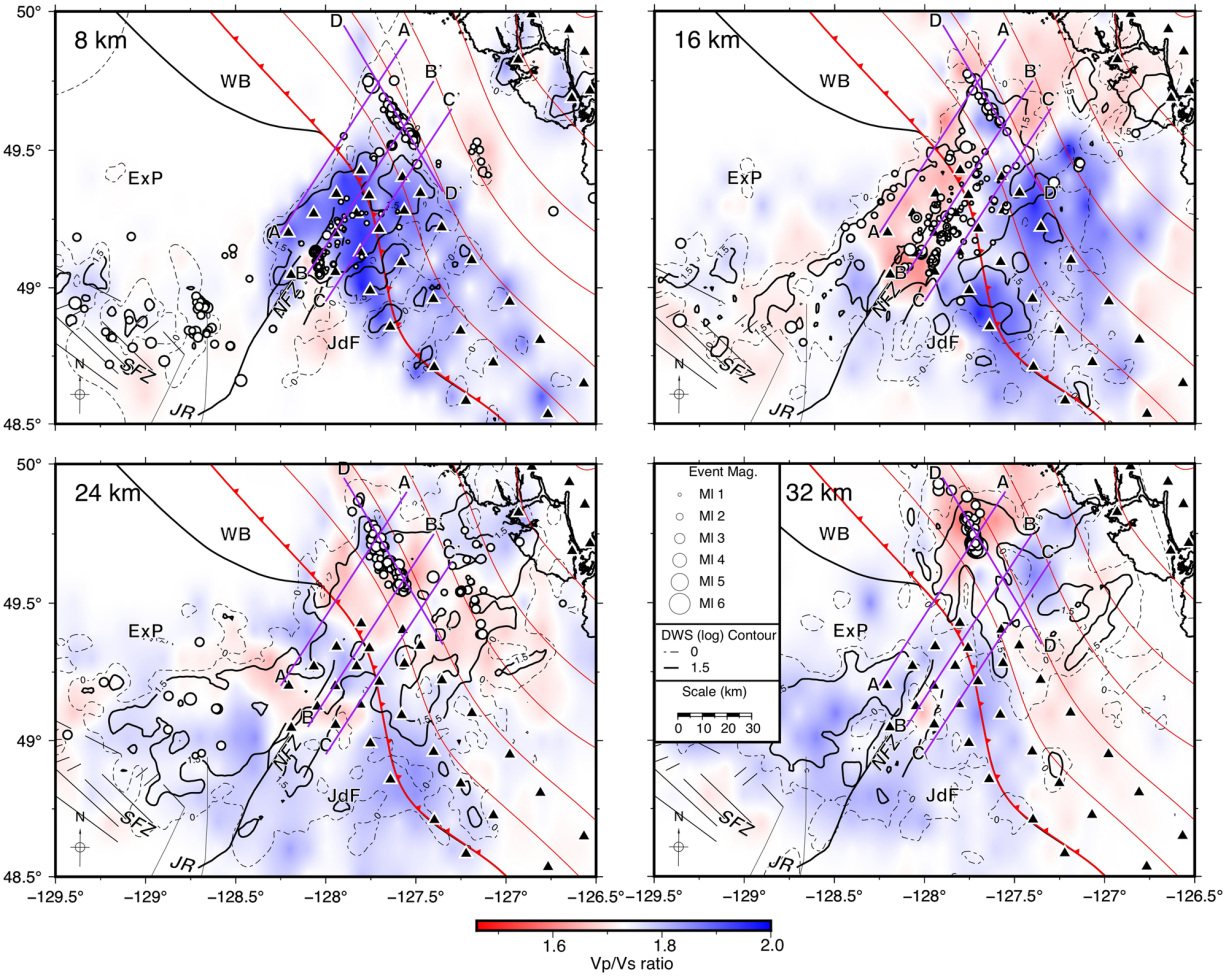
Depth slices of V_p_/V_s_ seismic tomography, generated with GMT^37^, at the same depths as Fig. 3. V_p_/V_s_ ratios greater than or less than 1.73 are shown as blue and red, respectively. DWS contours illustrate areas of high raypath densities and typically indicate where the tomography is best resolved. See Figs. 1 and 3 for the explanation of other features on the map.

The 3-D seismic tomography indicates both low- and high-velocity anomalies, defined as regions in which *P*-velocities, calculated over 1-km depth slices, are lower or higher than the average velocities of the 3D model within each depth slice by at least 2%. In Fig. 3, depths of 8, 16, 24, and 32 km are selected to focus on the shallower oceanic crust and oceanic mantle seaward of the subduction front, and the deeper oceanic crust and oceanic mantle landward of the subduction front. Further, V_P_/V_S_ ratios, presented in Fig. 4, can indicate differences in structure and rheology to further corroborate features observed from the *P-*velocity tomography.

Landward of the subduction front, where the Nootka Sequence occurred, a low-velocity anomaly is present at a shallow depth of ~ 8 km at approximately 49.5° N 127.3° W. This anomaly extends toward the northwest to greater depths with some localized variations (Fig. 3). A high-velocity anomaly is adjacent to the low-velocity anomaly to the southeast. We infer that the low-velocity anomaly represents the oceanic crust of the subducting ExP, and that the high-velocity anomaly is the oceanic mantle. At the depth of 8 km, the SeaJade I results show a continuous low-velocity anomaly within the NFZ^6^, which is consistent with the results from the combined dataset of both SeaJade I and II (Fig. 3). The better coverage and distribution of SeaJade II stations around the NFZ, as well as the greater number of earthquakes over a longer period of time, allow for a more accurate and detailed tomography model, compared to a model based just on SeaJade I data. The updated tomography results show that low-velocity anomalies within the southwestern NFZ and landward of the NFZ appear to be separated by a high-velocity anomaly over a distance of approximately 30 km. This high-velocity anomaly is particularly apparent at depths of 16 and 24 km (Fig. 3). Detailed tomography maps from depths of 20 to 31 km, the presumed depth range of the oceanic Moho discussed in Hutchinson et al.^6^, are shown in Figure S3.

In general, high-velocities and high V_P_/V_S_ ratios east of the NFZ, within the Juan de Fuca plate, are consistent with the SeaJade I results^6^. Within the Explorer plate immediately west of the NFZ, seismic velocities appear to be higher than average, and V_P_/V_S_ appears to be slightly lower at the depths of 16 to 24 km (Figs. 3 and 4).

**Figure 5:**
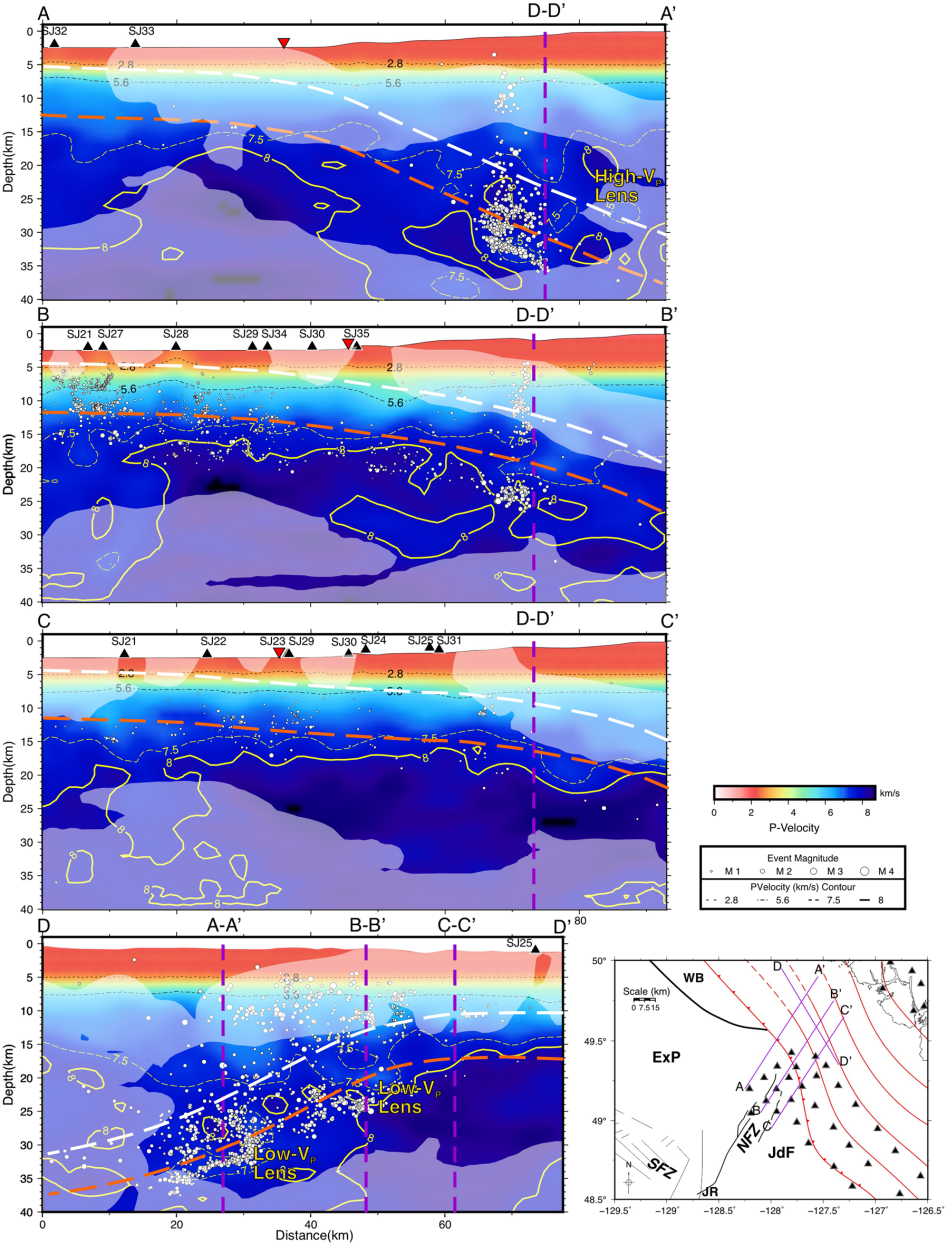
Cross-section profiles of Vp seismic tomography, labelled A-A' through D-D', generated with GMT^37^. Earthquakes within 5 km of the profiles are projected and marked as white-filled circles, while stations within 10 km of the profiles are marked as black triangles. Vp contours are shown as back and yellow lines labeled with representative *P*-velocities. A reference map for the study area and the profile lines are shown in the lower right corner. Cross-section intersections are shown with dashed purple lines and are labelled accordingly. The approximate location of the subduction front in profiles (**A**–**C**) are indicated by the upside-down red triangles. White and orange dashed lines are representative of the inferred top of the oceanic plate and oceanic Moho, respectively, based on and modified for this study from Hutchinson et al.^7^ and Hutchinson et al.^6^. Translucent white overlays indicate areas of lower raypath density corresponding to the log value of the DWS.

**Figure 6:**
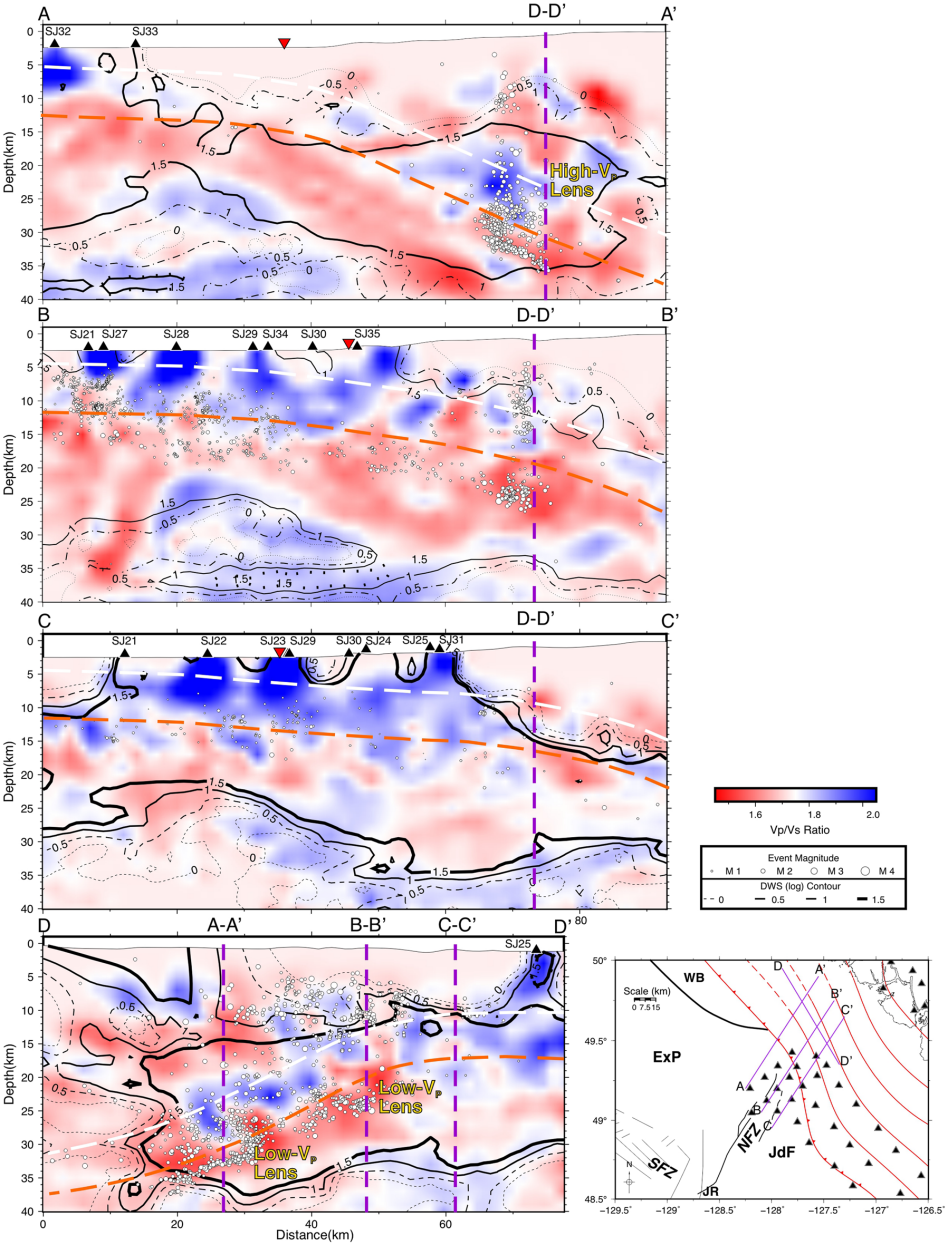
Cross-section profiles of V_P_/V_S_ ratio seismic tomography with raypath density DWS contours, generated with GMT^37^. The inferred Moho, shown as a dashed orange line, follows dipping low V_P_/V_S_ materials (< 1.65) overlain by high V_P_/V_S_ materials (> 1.75), which also coincide with the distribution of deep hypocentres in the subducting slab. See the Fig. 5 caption for additional information.

Several velocity profiles illuminate a change in the subducting slab geometry, from northwest to southeast (Fig. 5). Areas with lower raypath coverage are shown with translucent white overlays on the velocity profiles. To accompany these profiles, we show V_P_/V_S_ ratio tomography and derivative-weight sum contours of raypath densities with percent velocity perturbations from the original 1-D model (Fig. 6 and S4), which indicate areas with the best-resolved tomography. In regions where the V_P_ tomography results vary the most from the initial 1-D model, raypath densities are sufficiently high to indicate that the lateral variation of seismic velocity cannot be an artefact. Orange dashed lines in Figs. 5, 6, and S3 represent the oceanic Moho, which is interpreted from the general increase in depth of low-velocities and transitions from high (> 1.75) to low V_P_/V_S_ (< 1.75) ratios within the tomography model and the distribution of hypocentres. Merrill et al.^8^ attributed the sharp transition from high (> 1.75) to low (< 1.65) V_P_/V_S_ ratios with the Moho. From Hutchinson et al.^6^, the estimated seismogenic thickness of the uppermost oceanic mantle was found to be ~ 5–7 km, with a high concentration of earthquakes distributed near the crust-mantle interface. The depth to the top of the oceanic crust, indicated with dashed white lines in Figs. 5, 6 and S4 is extrapolated from the hypocentre distributions from this study and Hutchinson et al.^7^ and the assumed crustal thickness of ~ 7 to 8 km as determined in Hutchinson et al.^6^.

The 7.5–8.0 km/s *P*-wave velocity contours mark the transition from crust to uppermost mantle, i.e., the Moho discontinuity^18^. Within the oceanic plate seaward of the subduction front, the 7.5 and 8 km/s contours are nearly horizontal, parallel, and separated by a depth interval varying from ~ 2 km (Profile C–C′) to ~ 7 km (Profile A–A′; Fig. 5). This is indicative of a nearly uniform, near-horizontal geometry of the Moho discontinuity seaward of the subduction front separating the lower crust and uppermost mantle. To the southwestern extent of profiles A–A′, B–B′ and C–C′ (~ 0–20 km), the depth difference between the 7.5 and 8 km/s contours becomes significantly enlarged. This difference may represent the high degree of fracturing and-or deep mineral alteration of the upper mantle within the NFZ, as proposed by Rohr et al.^19^ and Hutchinson et al.^6^.

Past the subduction front, the 7.5 and 8 km/s contours diverge significantly. For example, along the northwesternmost profile (A–A′), located within the ExP, the slab is most likely more steeply dipping, since the 8 km/s contour goes from a depth of ~ 18 km to the lower limit of the tomography model at a depth 40 km (Fig. 5). The geometry of the slab in this region is not well determined from the *P*-velocities. However, we can estimate the dip of the subducting ExP as ~ 23° based on the distribution of hypocentres and V_P_/V_S_ ratio tomography. Northeast of the intersection of profile D–D′ with A–A′, a lens of high-velocity material overlies lower-velocity materials at a depth of approximately 20 km.

Within the NFZ, the velocity structure becomes more complicated on the landward side of the subduction front (profile B–B′, Fig. 5). At 45–70 km along profile B–B′, a significant low-velocity anomaly is present at a depth of approximately 25–33 km. This anomaly occurs beneath the subduction front, and it appears to continue (with less certainty) in profile A–A′. At present, we do not have an explanation for this feature. Continuing along profile B–B′ at approximately 70 km, the 7.5 km/s contour within the overriding plate appears to be deeper toward the northeast with several localized small-scale velocity variations. Similarly, the 8 km/s contour occurs at a depth of ~ 25 km at a model distance of 80 km along profile B–B′ (Fig. 5). The change in contour depths, along with the dipping low V_P_/V_S_ structure (Fig. 6), are compatible with a subducted oceanic slab dipping at ~ 17°.

Further southeast, within the JdF, the dip of the subucting slab appears to be even shallower, although it is difficult to estimate the dip of the slab without a more robust downdip hypocentre distribution. However, based on the V_p_ and V_P_/V_S_ tomography (profile C–C′ in Figs. 5 and 6), the 7.5 km/s contour landward of the subduction front between the distances of 65 and 85 km shows an estimated dip of 13–15°.

Across profile D–D′ (Fig. 5), seismic velocities vary drastically at depths below 15 km. Southeast of the intersection with profile B–B′, V_P_ is generally higher than to the northwest. Low-velocity materials (< 7.5 km/s) are located at approximately 20 to 50 km along profile D–D′ at depths of ~ 30 km to 17 km. This low-velocity structure is highlighted particularly well by perturbations from the 1-D velocity model in Figure S4.

Seaward of the subduction front, velocities within the NFZ are higher than the initial 1-D velocity model. At a depth of ~ 16 km, V_P_ generally exceeds 8 km/s (Fig. 3). This indicates that the upper mantle is located shallower within the NFZ near the subduction front than in the ExP, where V_P_ is less than 8 km/s at the same depth. We interpret the depth to the Moho within the NFZ to be ~ 13 km, where velocities exceed 7.5 km/s. In comparison, the oceanic crust of the ExP appears thicker (Fig. 5, profile A–A′), potentially extending to the depth of 15 km with a less well-defined Moho than along the NFZ and JdF tomography (profiles B–B′ and C–C′, respectively).

### Focal mechanism patterns

**Figure 7:**
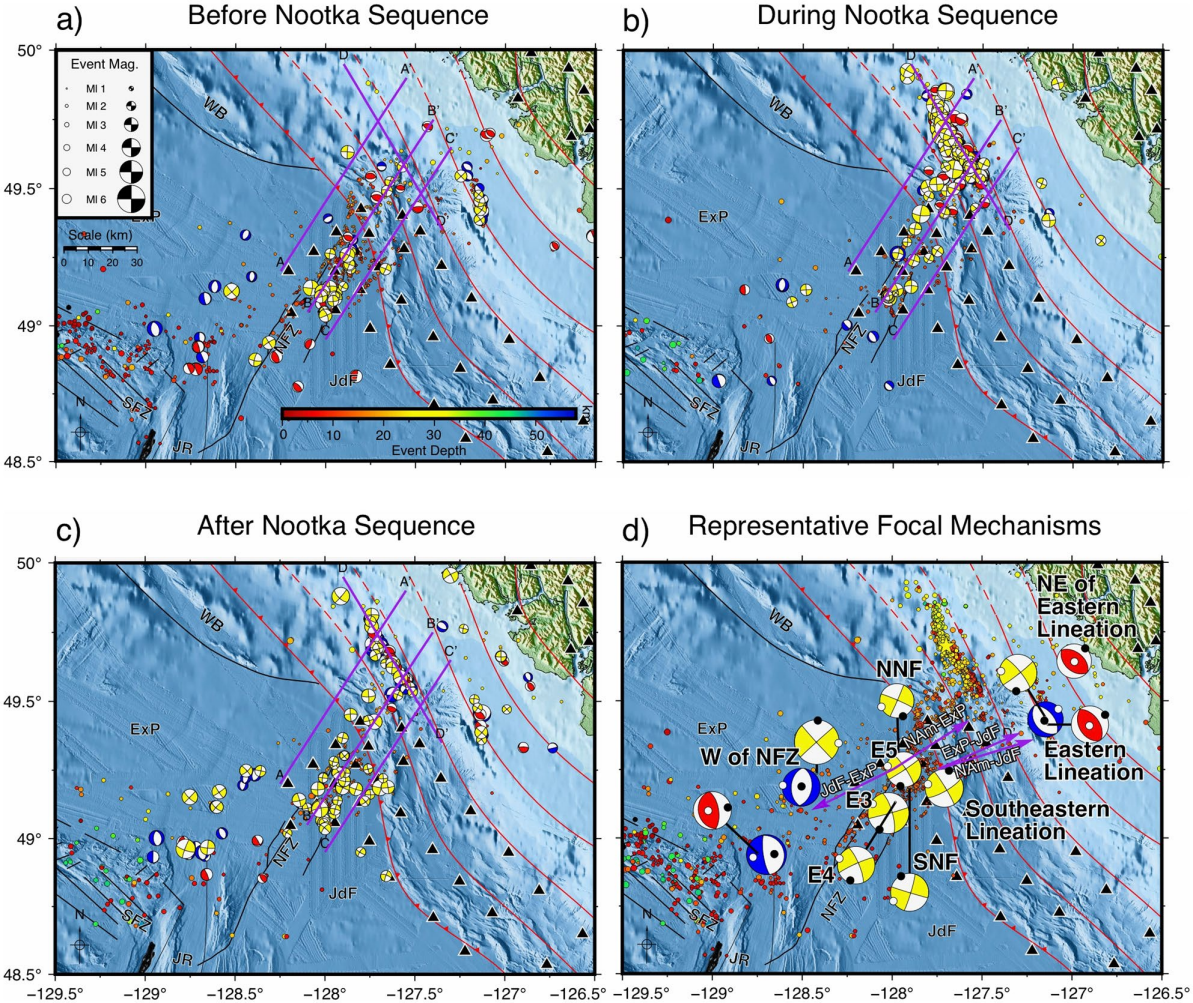
Maps of focal mechanism solutions, generated with GMT^37^, from before (**a**), during (**b**), and after (**c**) the Nootka Sequence. Representative averaged focal mechanism solutions from all three time windows are shown in (**d**) and are given in Table 2. The sizes of the focal mechanisms are indicative of M_L_ while colour represents rupture type; red = reverse, blue = normal, yellow = strike-slip. The purple arrows in (**d**) show relative plate motions between pairs of plates; the labels indicate the motion of the second plate relative to the first. Plate motions involving the ExP were calculated with model A of Braunmiller and Nábělek^22^, while all others were calculated with NUVEL-1A. *ExP* Explorer plate, *JdF* Juan de Fuca plate, *SFZ* Sovanco fracture zone, *NFZ* Nootka fault zone, *JR* Juan de Fuca Ridge, *WB* Winona Block. Representative focal mechanisms sizes have a scale 3 times that in the other diagrams. See Fig. 1 for the explanation of other features on the map.

Focal mechanisms from before, during, and after the Nootka Sequence are shown in Fig. 7a–c, respectively. We focus on the normal mechanisms to the west of the NFZ, reverse mechanisms to the northeast of the Nootka Sequence fault, and strike-slip mechanisms along the primary and secondary faults of the NFZ.

To better visualize the seismotectonic characteristics of individual structures, we provide representative focal mechanism patterns for areas of interest in Table 2 and show them in Fig. 7d. We use the FMC code adapted from Álvarez-Gómez^20^. Specifically, we first compute the Aki-Richards^21^ moment tensor components from the strike, dip, and rake for focal mechanisms grouped by rupture type and geographic setting. From these components, we then average and calculate the P-, T-, and B-axes of spatially grouped events with similar rupture types from the entire study. Finally, we compute the corresponding strike, dip, and rake for representative focal mechanisms from the averaged P-, T-, and B-axis vectors.

**Table 2:** Representative average focal mechanism solutions for selected areas.

Zone	Strike (°)	Dip (°)	Rake (°)	Aux-Strike (°)	Aux-Dip (°)	Aux-Rake (°)	P-axis Trend (°)	P-axis Plunge (°)	T-axis Trend (°)	T-axis Plunge (°)	Type^e^
W of NFZ^a^	9.6	43.7	− 84.6	182.2	46.6	− 95.1	27.4	86.0	275.8	1.5	N
W of NFZ (further SW)	171.1	76.9	− 86.7	336.7	13.6	− 104.0	85.6	58.0	258.4	31.8	N
NE of Eastern Lineation^b^	308.3	47.3	86.5	133.4	42.8	93.8	40.7	2.2	171.6	86.6	R
Eastern Lineation	51.6	85.8	10.8	320.8	79.2	175.8	185.8	4.7	276.7	10.6	S
Eastern Lineation	142.7	37.0	− 87.8	319.9	53.0	− 91.7	221.6	81.9	51.1	8.0	N
Eastern Lineation	331.3	53.6	97.6	138.7	37.1	79.9	55.9	8.3	272.7	79.7	R
SE Lineation	59.8	76.9	4.9	328.7	85.2	166.8	14.9	5.8	283.6	12.7	S
NNF	23.7	88.6	3.8	293.6	86.2	178.6	158.6	1.7	248.7	3.7	S
SNF	16.7	85.0	− 1.4	106.8	88.6	− 175.0	331.9	4.6	241.7	2.5	S
E3	74.4	88.0	15.2	343.9	74.8	178.0	208.2	9.3	300.2	12.1	S
E4	245.8	83.7	− 12.3	337.1	77.8	− 173.6	201.0	13.1	291.9	4.1	S
E5	56.4	86.5	9.4	325.8	80.7	176.5	190.8	4.1	281.4	9.1	S
Relict Slip Zone^c^	227.9	89.0	1.8	137.8	88.2	179.0	2.8	0.6	92.9	1.9	S
Western Reverse Faults^d^	351.1	62.6	92.9	164.8	27.5	84.4	78.9	17.6	267.9	72.2	R

^a^Located within the vicinity of 49.15° N, 128.5° W.

^b^Located within the vicinity of 49.7° N, 127° W.

^c^Located within the vicinity of 49.25° N, 128.5° W.

^d^Located within the vicinity of 48.8° N, 128.75° W.

^e^Rupture classification type is labelled as follows: *N* normal, *R* reverse, *S* strike-slip.

The NFZ is dominated by strike-slip mechanisms both before and after the Nootka Sequence. At 49.25° N, 128° W, the approximate motion of the ExP relative to the JdF is 219.35° from north at a speed of 2.5 cm/yr according to Model B of Braunmiller and Nabelek^22^, resulting in pure strike-slip relative motion between the two plates. Their Model A has a slight oblique-extensional component with a direction of 243.12° at 2.3 cm/yr. By comparison, the average trend of the T-axes for the northwestern Nootka fault (NNF) and southeastern Nootka fault (SNF) are in directions of 248.7° and 241.7°, respectively, which are more consistent with their Model A.

Normal-faulting mechanisms west of the NFZ, within the ExP at approximately 49.15° N, 128.5° W, have strikes aligned roughly north–south. The average trend of the T-axes for the normal mechanisms is 275.8° (or 95.8°). Similarly, T-axes of nearby strike-slip events have an average trend of 272.9° (or 92.9°). The orientation of the T-axes is counter-clockwise to the relative motion between the ExP and JdF. In a regional context, distributed deformationcan lead to complex rupture sequences, with variations in stress direction and magnitude over short distances. The presence of normal-faulting mechanisms is understood to reflect such stress heterogeneities.

Reverse mechanisms located above the subduction interface to the NE of the subduction front at approximately 49.7° N, 127° W are consistent with margin-normal compression caused by the subducting plate across the plate interface. The trend of the average P-axes of these focal mechanisms (40.7°) more closely matches the motion of the ExP with respect to NAm (2.1 cm/yr in a direction of 42.2°^22^) than that of JdF (3.8 cm/yr in a direction of 60.8°) with NUVEL-1A^23^.

## Discussion

### Northwestward bending and deformation of the shallow subducted Explorer plate

The hypocentre distribution of the Nootka Sequence demonstrates variation in the geometry of the shallow subducted ExP as described in Hutchinson et al.^7^ which agree with the deformation further downdip observed by Audet et al.^24^. From our prior study, it is proposed that toward the northwest, along the Nootka Sequence fault, the depth to the oceanic Moho increases from ~ 22 to 35 km (Fig. 5) over a distance of 25 km. The change in Moho depth indicates a significant bend or tear in the ExP nearly perpendicular to the direction of subduction. Further, Merrill et al.^8^ have proposed that the NFZ is acting as a block independent of the JdF plate, potentially separated by a downdip slab tear.

In this study, we observe lower-velocity materials with increasing depth from the southeast to the northwest along profile D–D′ (Fig. 5) from depths of ~ 15 km to greater than 30 km. This change in *P*-velocity is representative of the overall change in the geometry of the subducting slab. The presence of deep low-velocity materials supports the distribution of hypocentres as indicators of northwestward bending of the shallow subducted ExP. Further, the transition from high to low V_P_/V_S_ ratios is indicative of the depth to the Moho (Fig. 6), providing support for our inferred plate geometry and were not previously observed by Merrill et al. (2022)^8^.

Bending and unbending in the subducting plate toward the northwest are the most likely processes to create fractures, resulting in low velocities, not unlike the observations made for the deep low-velocity anomalies within the Nootka fault zone discussed in Hutchinson et al. (2019). The presence of low-velocity anomalies at ~ 30 km and ~ 70 km along profile D–D′ and depths of ~ 30 km and ~ 17 km, respectively, could be magnified because of this high degree of fracturing. These velocity features are particularly evident in Figure S4. We attribute the low-velocity anomalies as the likely signature of the northward dipping and bending ExP slab.

Past 80 km along profile A–A′, a lens of high-velocity materials is sandwiched between lower-velocity materials. Presumably, this lens could be mantle materials overlaying the subducted slab, either the upper mantle of the overriding plate or oceanic mantle emplaced by margin-parallel mantle flow around the slab’s edge. Shear-wave splitting analysis has indicated margin-parallel mantle flow in this region^25^, but whether it is induced at the edge of the subducted ExP further north, or by a more local intraslab tear^7^, as has been attributed to the fragmenting Cocos plate^26^, remains unknown.

On the JdF side of the profile D–D′, it appears that that the depth to the Moho (~ 15 km) is even shallower to the southeast of profile C–C′ (Fig. 5). We were previously unable to determine this change in plate geometry from the distribution of hypocentres alone, as they are sparse in this region^7^. If any slab tearing is present between the JdF and NFZ, as proposed by Merrill et al.^8^, it would have to occur further downdip, as no sharp offsets in velocity southeast of profile C–C′ that can be observed along profile D–D′ (Fig. 5).

### Tectonic evolution of the NFZ

Faults from outside of the NFZ may be representative of a time when shearing was less concentrated along the mature NNF and SNF^19^. The decrease in the length from the Nootka Sequence (~ 60 km) to the lineations that delineate the modern secondary conjugate faults (~ 10 km) between the northwestern and southeastern primary faults, probably implies that the NFZ has matured by changing from a more broadly distributed shear zone to a concentrated zone with well-defined bounding faults. The Nootka Sequence fault itself has also presumably grown and matured with time, as hypocentres are located within the oceanic mantle, similar to the northern and southern primary faults^7^, and unlike the conjugate faults within the NFZ^6^.

**Figure 8:**
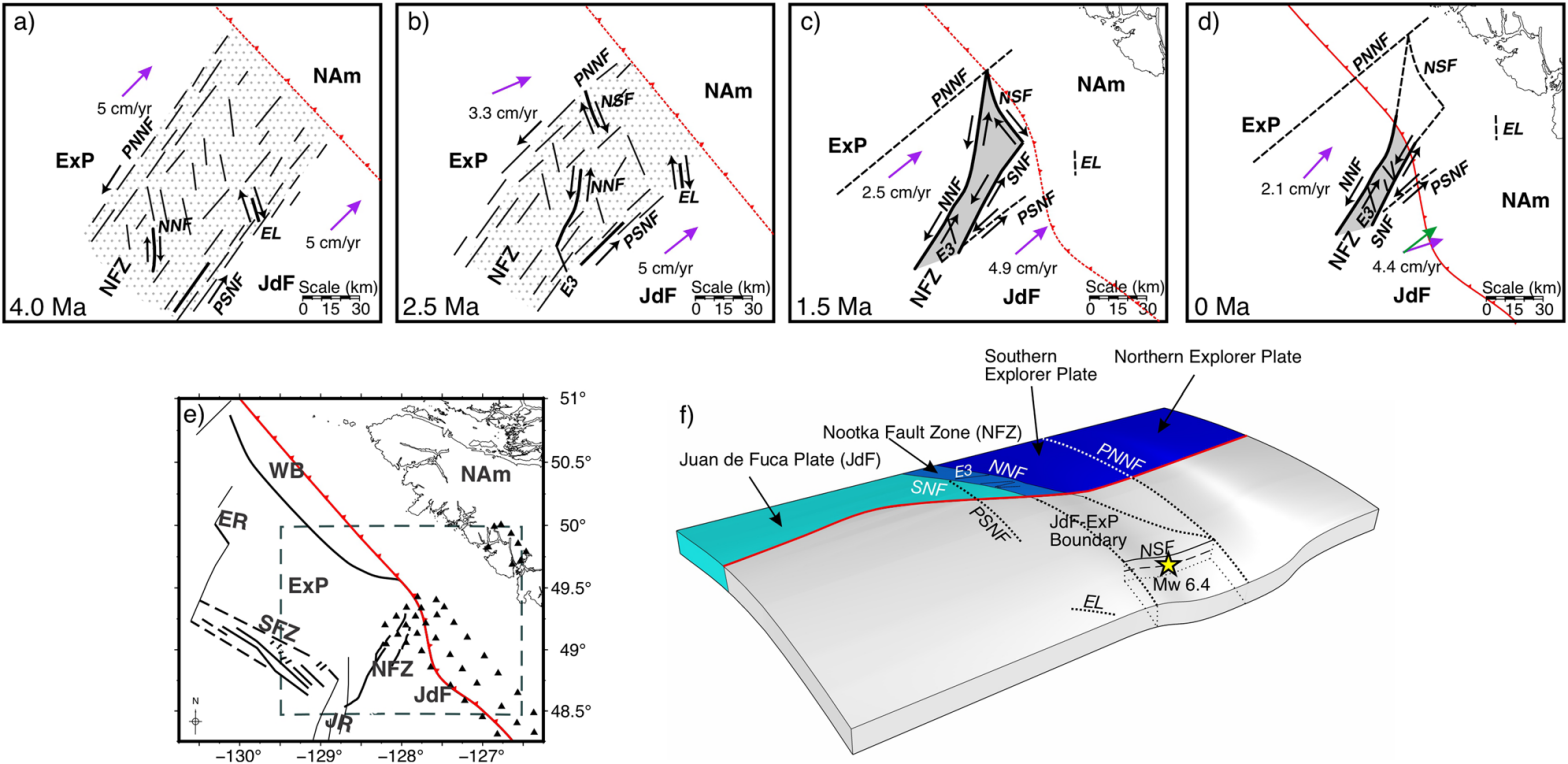
Illustrative diagram of the presumed evolution of the Nootka fault zone from 4.0 Ma (**a**) to 0 Ma (**d**). (**e**) shows a map of the current configuration the tectonic plates, ridges, and fault zones in the study area. The dashed grey box shows the bounds of (**a**–**d**). At 4.0 Ma (**a**), the Nootka fault zone begins as a region of distributed shear between the Explorer and Juan de Fuca plates. Clockwise rotation occurs between ~ 3.5 Ma and 1.5 Ma. The subduction front has presumably developed with the deposition of sediments, so the exact configuration prior to 0 Ma is unknown. Relative motions along faults are indicated by opposing black arrows. Areas of distributed shear are indicated by gray stippling. Solid gray regions indicate that shear dislocation only happens along individual faults. Purple arrows show the relative motion of the Explorer and Juan de Fuca plates relative to North America, derived from^22,28^. The green arrow shows our estimated relative motion of the Juan de Fuca plate relative to North America derived from reverse mechanism P-axes. (**f**) shows a 3-D illustrated diagram of the current plate configurations, updated from Hutchinson et al.^7^. Text labels are abbreviations for the following features: *ExP* Explorer plate, *JdF* Juan de Fuca plate, *NFZ* Nootka Fault Zone, *PNNF* paleo-northwestern Nootka fault, *PSNF* paleo-southeastern Nootka fault, *NNF* northwestern Nootka fault, *SNF* southeastern Nootka fault, *EL* eastern lineation, *NSF* Nootka Sequence fault, *NAM* North America plate, *JR* Juan de Fuca Ridge, *ER* Explorer Ridge, *WB* Winona Basin.

A lineation southeast of the SNF (Figs. 1 and 2; Table 1) may have formed at a time before the NFZ underwent a period of rotation. This lineation appears rotated clockwise by approximately 11° relative to the current NNF and 25° relative to the SNF. Another lineation observed east of the Nootka Sequence (Fig. 1; Table 1), landward of the subduction front, also appears rotated clockwise by approximately 17° relative to the average strike of the NFZ conjugate faults and the Nootka Sequence (156°). Davis and Riddihough^27^ have demonstrated that the Explorer Ridge migrated to the northwest with the isolation of the Winona Block from the Pacific plate from ~ 4 to 1 Ma (Fig. 8). The current position of the Winona Block is represented by a basin to the northwest of the NFZ, while the Explorer ridge is located to the northwest of the NFZ, marking the northern terminus of the Explorer plate. As migration of the Explorer Ridge occurred, clockwise rotation of the Explorer Ridge and the ExP reduced the rate of margin normal subduction.

In Fig. 8a–d, we present an illustrated evolution of the NFZ based on the rotation of seismic lineations and plate reconstructions from previous authors^19,22,27,28^, expanding on the recent proposals of Savard et al.^29^. While Savard et al.^29^ and Merrill et al.^8^ indicate that seismic lineations east of the NFZ within the subducted slab are part of the JdF, we propose that they are paleo-faults related to the NFZ. Figure 8e shows a map of the current tectonic configurations of the region and Fig. 8f presents an improved 3-D interpretation of the downgoing JdF and ExP plates in northern Cascadia from Hutchinson et al.^7^. We interpolate the velocity vectors for relative plate motions between the ExP and JdF plates using the rotation poles from Riddihough^28^ from ~ 4.0 Ma to the present with Model A as proposed by Braunmiller and Nábělek (2002)^22^.

#### 4 Ma

At 4 Ma, the ExP subducted much like the northern JdF at a rate of ~ 5 cm/yr nearly perpendicular to the subduction front^28^ at an orientation 15–25° counter-clockwise to what is presumed today^22^. The exact configuration of the subduction front at this time is unknown, so it is portrayed as a straight dashed red line (Fig. 8a). The oceanic lithosphere of the easternmost ExP is ~ 0.5 Myr old. The early NFZ formed at ~ 4 Ma because of the partial capture of the Winona Block from the Pacific plate by NAm, and it is portrayed as a broad deformation zone with many smaller immature faults. The boundaries of the NFZ, the paleo-northwestern Nootka fault (PNNF) and paleo-southeastern Nootka fault (PSNF), are based on the northern terminus of the Nootka Sequence Fault (NSF) and the location of several strike-slip events to the northwest of the current NFZ and the Southeastern Lineation, respectively. We have inferred that the current northern primary fault, demarcated as the northwestern Nootka fault (NNF), may have initiated as a conjugate fault during this time along with the Eastern Lineation, based on their paleo-orientations.

#### 2.5 Ma

Clockwise rotation of the Pacific plate and the Explorer Ridge relative to NAm has led to partial capture of the Pacific plate by NAm. This capturing initialized the formation of the Winona block, reducing the convergence rate of the ExP. Due to rotation of the ExP and continued rupturing and evolution of the NFZ, we speculate that the NSF began as a conjugate fault (Fig. 8b). The NNF continues to mature and lengthen nearly parallel to the direction of subduction. Subduction of the Eastern Lineation begins shortly before 1.5 Ma^27^.

Development of the most well-defined conjugate fault (E3, Figs. 2 and 7) may have begun during this time. The more northerly orientation in comparison to the other conjugate faults indicates that it may have undergone some rotation, and its well-defined lineation is suggestive of an older, more mature fault.

#### 1.5 Ma

As the ExP plate subducts, the angle of subduction relative to NAm is more northward when using the rotation poles from Riddihough (1984)^28^ (Fig. 8c). Maturation of the NFZ has led to the formation of the current SNF and lengthening of the NSF, bridging the distance between the NNF and SNF. The NNF has further lengthened to the southwest and connected with the NSF to the north. As plate motion was accommodated by the narrower NFZ, the PNNF and PSNF were abandoned as the primary active boundaries of the NFZ. Subduction of the NSF has begun.

Further northwest, at ~ 1 Ma, the Winona block is isolated from the Pacific plate. The subduction front may more closely resemble the present-day configuration, but it is still portrayed with a dashed red line to account for uncertainty.

#### 0 Ma

The azimuthal difference between the orientation of paleo-faults and that of currently primary faults (e.g., the orientation of the Southeast Lineation relative to the SNF) indicates an average of 18° counter-clockwise rotation (Fig. 8d). Since 3.5 Ma the NFZ has matured and narrowed. The current NNF and SNF extend well into the mantle, acting as potential conduits for hydration of mantle materials. Conjugate faults, particularly E3, have become well-developed between the NNF and SNF. Rupturing along paleo-faults continues to occur regularly, as they were first noted during SeaJade I by Hutchinson et al.^6^, but with less frequency than the current primary and secondary faults.

### Focal mechanisms and regional tectonics

"Focal Mechanism Patterns" section and Table 2 discuss focal mechanisms in the area surrounding the NFZ in relation to current regional tectonic models. The orientations of focal mechanisms from the NNF and SNF generally agree with the sense of motion between the JdF and ExP. Focal mechanism solutions orient well with the associated faults (Fig. 7d) and indicate left-lateral motion for the primary faults and right-lateral motion for the secondary conjugate faults. Low V_P_ and V_P_/V_S_ ratios within the NFZ verify the results from SeaJade I^6^ and lend further support to the extreme fracturing and altering of the lower oceanic crust and upper mantle proposed by Rohr et al. (2018).

Outside of the NFZ, plate motion vectors inadequately explain the more northerly orientation of P-axes from reverse mechanisms northeast of the NSF. Because the averaged P-axes of the reverse mechanisms are nearly identical to the sense of motion between the NAm and ExP (Fig. 7d), we propose that the northern JdF is subducting nearly 20° counter-clockwise to the relative plate motions as determined with the NUVEL-1A model^23^ (our relative motion direction is shown as a green arrow in Fig. 8d). Brothers et al.^30^ calculated a new Euler rotation pole for the interaction between the Pacific plate and the NAm by analysis of morphological features and reconstructions of fault offsets along the Queen Charlotte fault. They proposed that relative motions between the two plates near the southern Queen Charlotte fault have an obliquity of 7.4°, which results in almost purely strike-slip behaviour, as opposed to having a stronger compressional component with an obliquity of 18.3°, as reported by DeMets et al. (2016)^31^. If so, this demonstrates that relative plate motions for both the JdF^23,32^ and ExP^22^ may require re-evaluation, as they have both depended on the interaction between the Pacific and NAm plates.

An abundance of normal mechanisms within the ExP to the west of the NFZ indicates a locally dominant tensional stress field. Given the contrast between subduction of the southern ExP and the various interpretations of the Winona block: no subduction^33,34^, subduction^27^, or transpression^35^, it is likely that the ExP is not acting as a single contiguous plate. Or instead, the ExP could be in the process of reconfiguration. These mechanisms can be explained by regional shearing, where the T-axes align with σ3. The normal mechanisms appear to line up with the strike of the Juan de Fuca Ridge when following its trace from the south (Fig. 7d). As an alternative to regional shearing, the normal mechanisms could be related to northward propagation of current seafloor spreading extension of the northern Juan de Fuca Ridge.

Our observed focal mechanism solutions, deformation of the shallow subducted ExP, and the continuous evolution of the NFZ as determined from the distribution of faults^19^ and lineations of hypocentres^6^ are indicative of an unstable and changing tectonic regime. We propose that direct observations of the ExP and JdF in northern Cascadia, such as with seafloor geodesy, is required for re-evaluation of relative plate motions. Further seismic network surveys north of the SeaJade I and II OBS sites could provide a more accurate assessment of the internal deformation of the fragmenting ExP.

## Conclusions

Three-dimensional seismic tomography in combination with our previous analysis of the hypocentre distribution of the Nootka Sequence^7^ confirms the downward bend in the ExP toward the northwest with plunging low-velocity and low V_P_/V_S_ structures representing the subducting plate. The estimated dip of the subducted plate just landward of the subduction front changes from 23° in the northwest to 13° in the southeast. Seaward of the subduction front, we estimate the depth to the oceanic mantle to vary between 13 and 15 km below sea level from the JdF to the ExP. Low V_P_/V_S_ ratios within the NFZ extend to depths of ~ 30 km, particularly near the secondary fault E3, likely indicating high degrees of fracturing, potential water infiltration and alteration, and localized shear deformation.

Averaged focal mechanism solutions represent localized stress orientations. To the southwest and west of the NFZ, roughly N-S strike oriented normal focal mechanisms provide evidence for a localized tensional stress field. Focal mechanisms within the NFZ confirm that it is controlled by strike-slip failure, aligning with the faults mapped by hypocentre distributions. Downdip of the subduction front, paleo-faults, such as the Nootka Sequence Fault and the Eastern Lineation (Fig. 7d), exhibit complex rupture, presumably due to competing tectonic forces in the deformation of the ExP. Finally, thrust mechanisms within the overriding NAm are likely produced as a product of convergence, while the average P-axes (~ 40°) better align with the plate motion vector of the ExP than JdF relative to NAm.

The hypocentre distributions and focal mechanism solutions indicate currently active faults within the NFZ and the orientations of local stresses. Lineations of hypocentres located further from the primary and secondary faults of the NFZ have orientations rotated at an average of 18° clockwise, with more broadly distributed seismicity. We interpret these lineations as paleo-faults, which are much less active than the NNF, SNF, and associated conjugate faults, at present. These faults may represent the residual signature of a broader, less mature NFZ that began formation nearly 4 Ma with the capture of the Winona block^27^.

Our observations provide direct evidence for extreme deformation of the ExP and indicate that current plate rotation models^22,23,32^ may not accurately capture the plate motions downdip of the subduction front. Further questions remain, such as how fragmented the ExP is, and where the limit of subduction is to the northwest. Addressing these questions in addition to our findings will lend to accurate seismic hazard prediction models for the northernmost extent of the Cascadia subduction zone.

## Supplementary Information

Supplementary Information.

## Data Availability

Seismograms used in this study were collected as part of the SeaJade II (Seafloor Earthquake Array—Japan Canada Cascadia Experiment, Phase II) project. Arrival data, relocated hypocentres, focal mechanisms, *P*-wave tomography, and *S*-wave tomography can be found in Supplemental Tables A1, A2, B1, C1, C2. Waveform data can be obtained from JAMSTEC upon request. The Natural Resources Canada—Earthquakes Canada database was searched using http://www.earthquakescanada.nrcan.gc.ca/stndon/NEDB-BNDS/bulletin-en.php. Some plots were made using the Generic Mapping Tools version 5.4.2 (http://gmt.soest.hawaii.edu/; Wessel and Smith 1998). Global Multi-Resolution Topography (GMRT) was used to generate high resolution topography and bathymetry for GMT maps^41^.
