# Supplementary material for: Tectonic evolution of the Nootka fault zone and deformation of the shallow subducted Explorer plate in northern Cascadia as revealed by earthquake distributions and seismic tomography
Source: Sci Rep. 2023 May 15;13:7873. doi: 10.1038/s41598-023-33310-z (PMC10185695; doi:10.1038/s41598-023-33310-z)
Supplement: Supplementary file 1 — Supplementary Information. [file 41598_2023_33310_MOESM1_ESM.docx]

Supporting Information for

**Tectonic evolution of the Nootka fault zone and deformation of the shallow subducted Explorer plate in northern Cascadia as revealed by earthquake distributions and seismic tomography**

Jesse Hutchinson^1,5^, Honn Kao^1,2^, Michael Riedel^3^, Koichiro Obana^4^, Kelin Wang^1,2^, Shuichi Kodaira^4^, Tsutomu Takahashi^4^, and Yojiro Yamamoto^4^

^1^ School of Earth and Ocean Sciences, University of Victoria, Victoria, BC, V8P 5C2, Canada

^2^ Pacific Geoscience Centre, Geological Survey of Canada, Natural Resources Canada, Sidney, BC, V8L 4B2, Canada

^3^ GEOMAR Helmholtz-Centre for Ocean Research Kiel, Kiel, Germany

^4^ Japan Agency for Marine-Earth Science and Technology (JAMSTEC), Yokohama, Japan

^5^ Now at Ocean Networks Canada, University of Victoria, Victoria, BC, V8P 5C2, Canada.

**Contents of this file**

Text SA to SC

Figures S1 to S3

Table S1

**Additional Supporting Information (Files available separately)**

Captions for Tables A1 to C2

**Introduction**

This document includes supporting information for supplemental tables, figures, and text. The supplemental tables A1, A2, B1, C1, and C2 include earthquake arrival, earthquake hypocentre, focal mechanism solutions, P-velocity tomography, and S-velocity tomography respectively. Figures S1 – S4 provide examples of typical seismic waveforms from this study, seismic tomography checkerboard test results, detailed P-velocity tomography maps, and P-velocity % perturbation tomography profiles. The supplemental text details information about the focal mechanism solutions and TomoDD procedures.

Text SA Earthquake Datasets

Data used in the earthquake arrival and hypocentre tables are available from the Open Science Forum at (<https://osf.io/5q9fb/?view_only=f67fbfee2011466ab879e547adb982fb>). Descriptions are detailed in the captions for tables A1 and A2.

Text SB Focal Mechanism Datasets

Focal mechanism solutions were computed with the program HASH (Hardebeck and Shearer, 2002, 2003). Like many programs used to calculate focal mechanism solutions (e.g. FPFIT; Reasenberg and Oppenheimer, 1985), HASH calculates the best-fit solutions for earthquakes from input *P*-arrival first motions. An additional benefit of HASH is that *S/P* amplitude ratios can be utilized to further constrain focal mechanism solutions. Theoretically, *P* amplitudes along nodal planes would be the smallest, while they would be the largest at the P and T axes. Therefore, *S/P* ratios are expected to be much larger along nodal planes than within focal sphere quadrants.

Several factors are utilized for determining the quality of a best-fit focal mechanism solution, which is ranked from A-F, with A being the best. Any focal mechanisms with fewer than 8 first motion polarities are given an F rank. Solutions with azimuthal gaps > 90° and takeoff angle gaps > 60° are given an E rank. Any solution with smaller gaps is considered at least a D-ranked solution. Higher ranks are given with smaller average misfits and RMS fault plane uncertainties, and larger station distribution ratios and focal mechanism probabilities. For a given earthquake, these values are determined from a set of focal mechanisms calculated over repeated trials. Ultimately, an A-ranked focal mechanism solution has an average misfit ≤ 0.15, an RMS fault plane uncertainty ≤ 25°, a station distribution ratio ≥ 0.5, a mechanism probability ≥ 0.8, a maximum azimuthal gap ≤ 90°, a maximum takeoff angle gap ≤ 60°, and 8 or more first-motion polarities.

The focal mechanism data described for the tables below are available from the Open Science Forum at (<https://osf.io/5q9fb/?view_only=f67fbfee2011466ab879e547adb982fb>). Descriptions are detailed in the captions for table B1.

Text SC Tomography Datasets.

TomoDD parameters from the TomoDD input file for the combined SeaJade I and II dataset are listed below. Note that the parameters are described in the documentation for both HypoDD (Waldhauser, 2001) and TomoDD (Zhang and Thurber, 2006). Descriptions for the TomoDD velocity model data tables are provided following the input parameter information.

*--- input file selection

* cross correlation diff times:

./new_dt_mincorr_08.cc

*

*catalog P diff times:

./new_dt.ct

* catalog absolute times

./new_absolute.dat

*

* event file:

./new_events.dat

*

* station file:

./new_station.dat

*

*--- output file selection

* original locations:

tomodd_seajade_02.loc

* relocations:

tomodd_seajade_02.reloc

* station information:

tomodd_seajade_02.sta

* residual information:

tomodd_seajade_02.res

* source paramater information:

tomodd_seajade_02.src

*Output velocity

tomodd_seajade_02.vel

* Vp model

Vp_model_02.dat

* Vs model

Vs_model_02.dat

*--- data type selection:

* IDAT: 0 = synthetics; 1= cross corr; 2= catalog; 3= cross & cat

* IPHA: 1= P; 2= S; 3= P&S

* DIST:max dist [km] between cluster centroid and station

* IDAT IPHA DIST

3 3 400

*

*--- event clustering:

* OBSCC: min # of obs/pair for crosstime data (0= no clustering)

* OBSCT: min # of obs/pair for network data (0= no clustering)

* OBSCC OBSCT CC_format

8 8 1

*

*--- solution control:

* ISTART: 1 = from single source; 2 = from network sources

* ISOLV: 1 = SVD, 2=lsqr

* NSET: number of sets of iteration with specifications following

* ISTART ISOLV NSET weight1 weight2 weight3 air_depth

2 2 18 15 15 15 -1.5

* i3D delt1 ndip iskip scale1 scale2 iuses

2 0 9 1 0.5 1.00 2

* xfac tlim nitpb(1) nitpb(2) stepl

1.3 0.0005 50 50 0.5

* lat_Orig lon_Orig Z_Orig iorig rota

49.25 -127.75 0 1 0

*

*--- data weighting and re-weighting:

* NITER: last iteration to used the following weights

* WTCCP, WTCCS: weight cross P, S

* WTCTP, WTCTS: weight catalog P, S

* WRCC, WRCT: residual threshold in sec for cross, catalog data

* WDCC, WDCT: max dist [km] between cross, catalog linked pairs

* WTCD: relative weighting between absolute and differential data

* THRES: Scalar used to determine the DWS threshold values

* DAMP: damping (for lsqr only)

* --- CROSS DATA ----- ----CATALOG DATA ----

* NITER WTCCP WTCCS WRCC WDCC WTCTP WTCTS WRCT WDCT WTCD DAMP JOINT THRES

5 0.1 0.05 -9 -9 0.5 0.25 -9 20 1 350 0 0.2

3 0.1 0.05 -9 -9 0.5 0.25 -9 20 1 350 1 0.2

5 0.1 0.05 -9 -9 0.5 0.25 -9 20 1 350 0 0.2

5 0.1 0.05 6 10 0.1 0.05 6 10 1 350 0 0.2

3 0.1 0.05 6 10 0.1 0.05 6 10 1 350 1 0.2

5 0.1 0.05 6 10 0.1 0.05 6 10 1 350 0 0.2

5 1.0 0.5 6 5 0.1 0.05 6 5 0.1 300 0 0.2

3 1.0 0.5 6 5 0.1 0.05 6 5 0.1 300 1 0.2

5 1.0 0.5 6 5 0.1 0.05 6 5 0.1 300 0 0.2

5 1.0 0.5 6 2 0.01 0.005 6 2 0.1 300 0 0.2

3 1.0 0.5 6 2 0.01 0.005 6 2 0.1 300 1 0.2

5 1.0 0.5 6 2 0.01 0.005 6 2 0.1 300 0 0.2

5 1.0 0.5 6 1 0.01 0.005 6 2 0.1 300 0 0.2

3 1.0 0.5 6 1 0.01 0.005 6 2 0.1 300 1 0.2

5 1.0 0.5 6 1 0.01 0.005 6 2 0.1 300 0 0.2

5 1.0 0.5 6 0.5 0.01 0.005 6 2 0.1 300 0 0.2

3 1.0 0.5 6 0.5 0.01 0.005 6 2 0.1 300 1 0.2

5 1.0 0.5 6 0.5 0.01 0.005 6 2 0.1 300 0 0.2

*

*--- event selection:

* CID: cluster to be relocated (0 = all)

* ID: cuspids of event to be relocated (8 per line)

* CID

1

* ID

Seismic tomography data tables are available from the Open Science Forum at (<https://osf.io/5q9fb/?view_only=f67fbfee2011466ab879e547adb982fb>). Descriptions are detailed in the captions for tables C1 and C2.


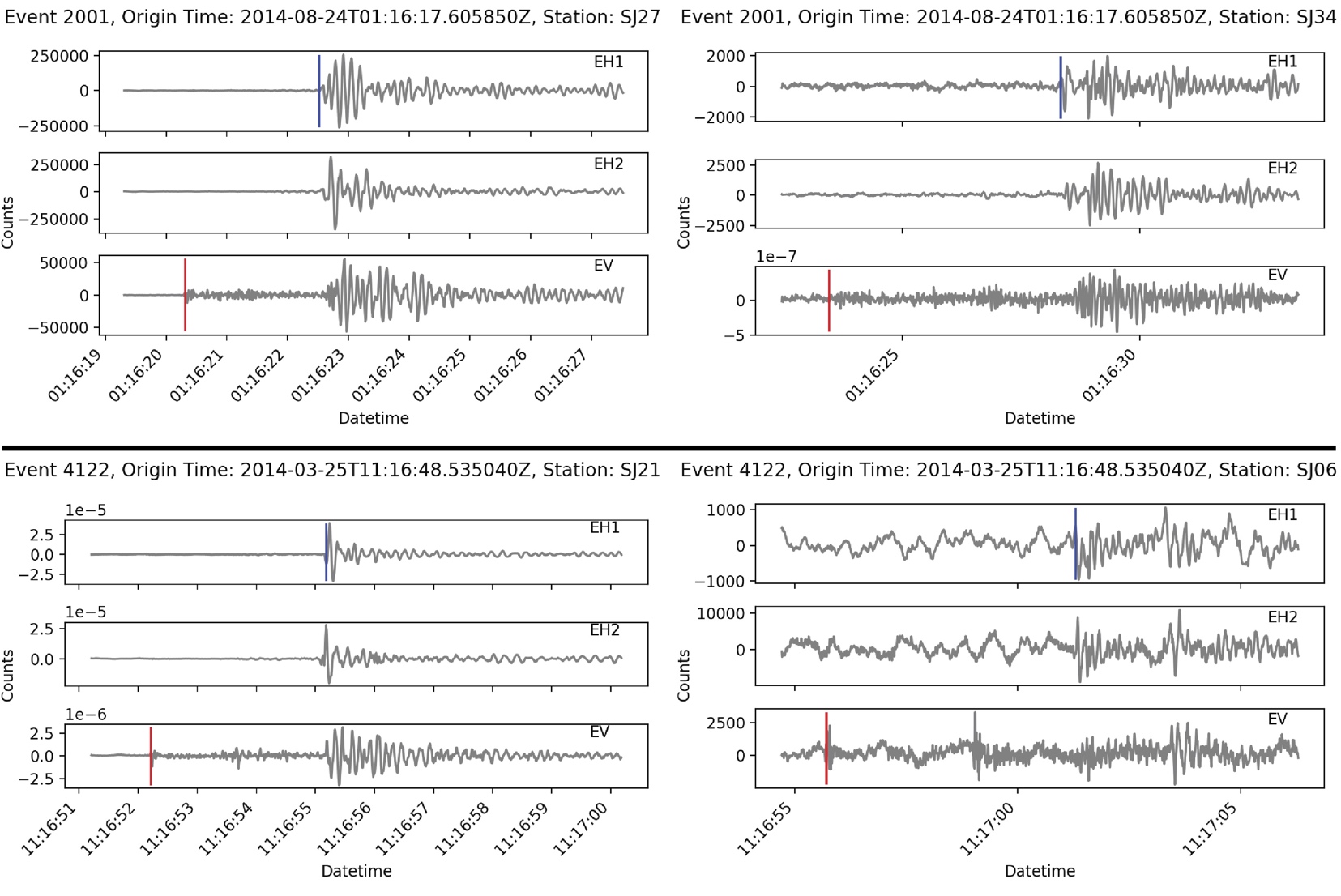


Figure S1. Seismic waveforms of data selected from two different events (2001 and 4122). These waveforms are typical of what was seen from both the SeaJade I and II datasets, and they are shown unfiltered or corrected for instrument response. For event 2001 (top), we show waveforms from stations SJ27 (left) and SJ34 (right). For event 4122 (bottom), we show waveforms from stations SJ21 (left) and SJ06 (right). Analyst determined P and S-phase arrivals are indicated by red and blue lines, respectively. The waveforms from Station SJ06 exemplify data more heavily contaminated by noise, while the waveforms from SJ34 show that P-phase arrivals are not always readily apparent.


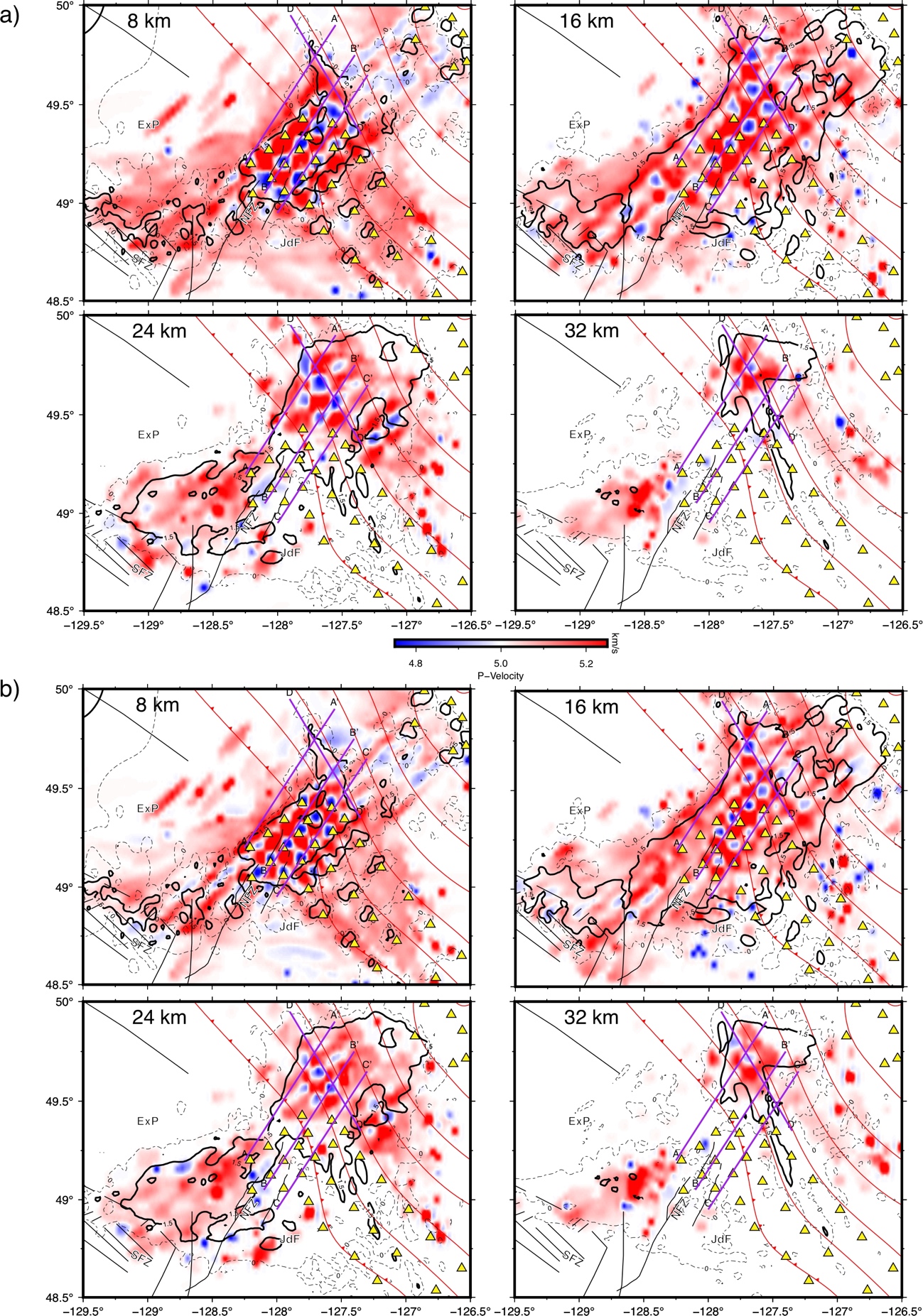


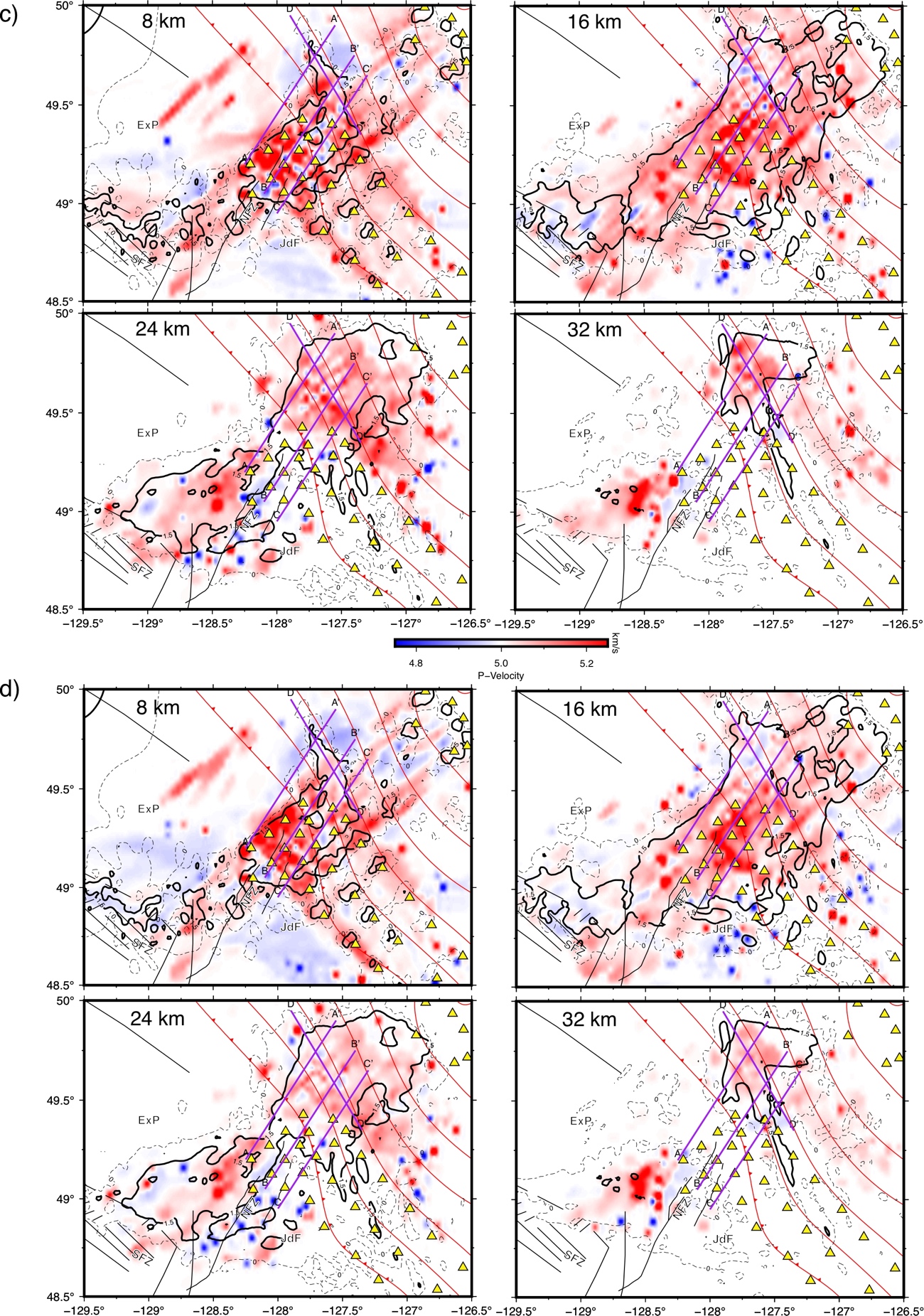


Figure S2. Maps of checkerboard tomography resolution tests

. Tests at depths of 8, 16, 24, and 32 km are shown for resolutions of a) 10-km, b) 8-km, c) 5-km, and d) 3-km.


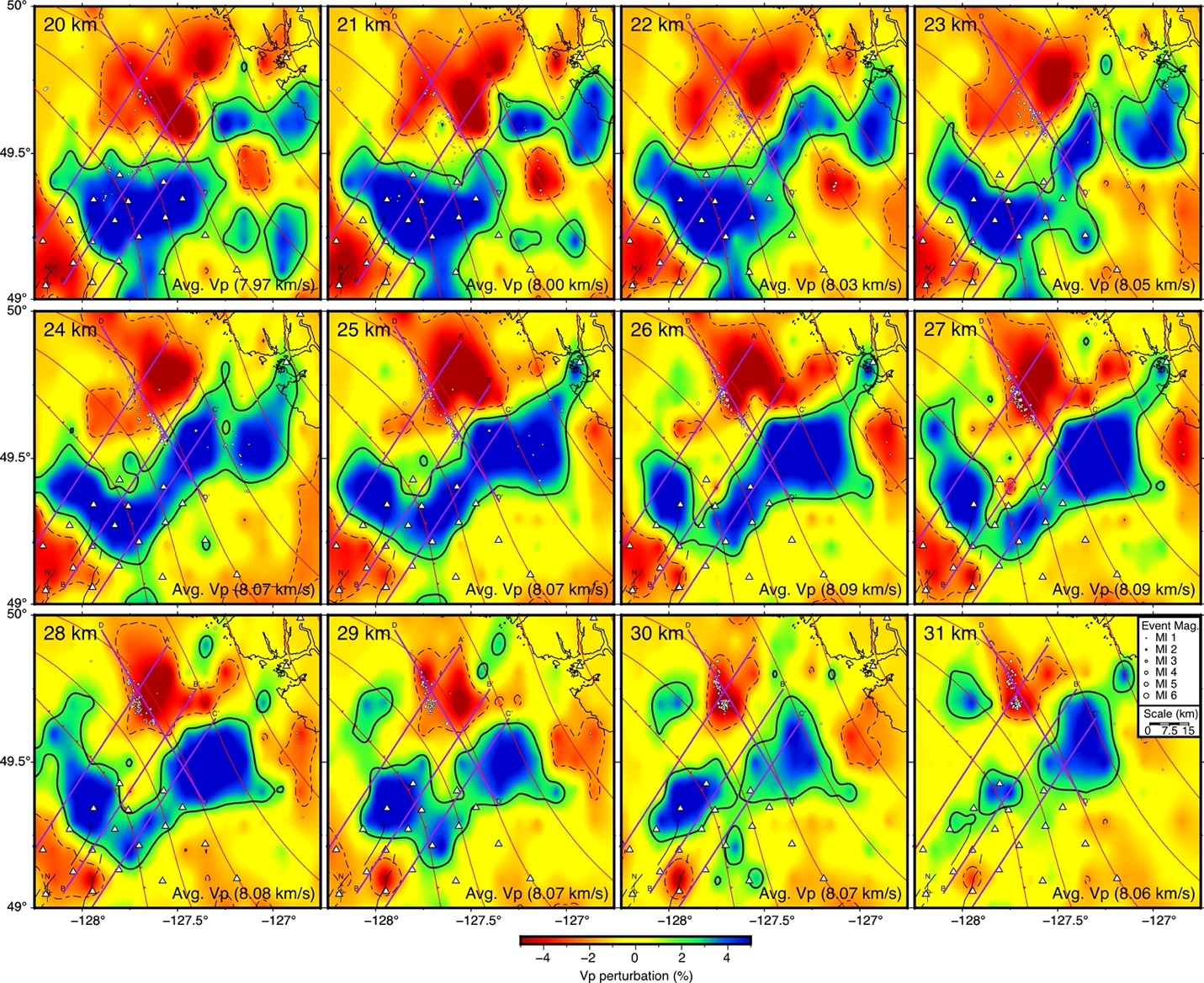


Figure S3. Detailed V_P_ seismic tomography depth slices focusing mainly on the region landward of the subduction front.


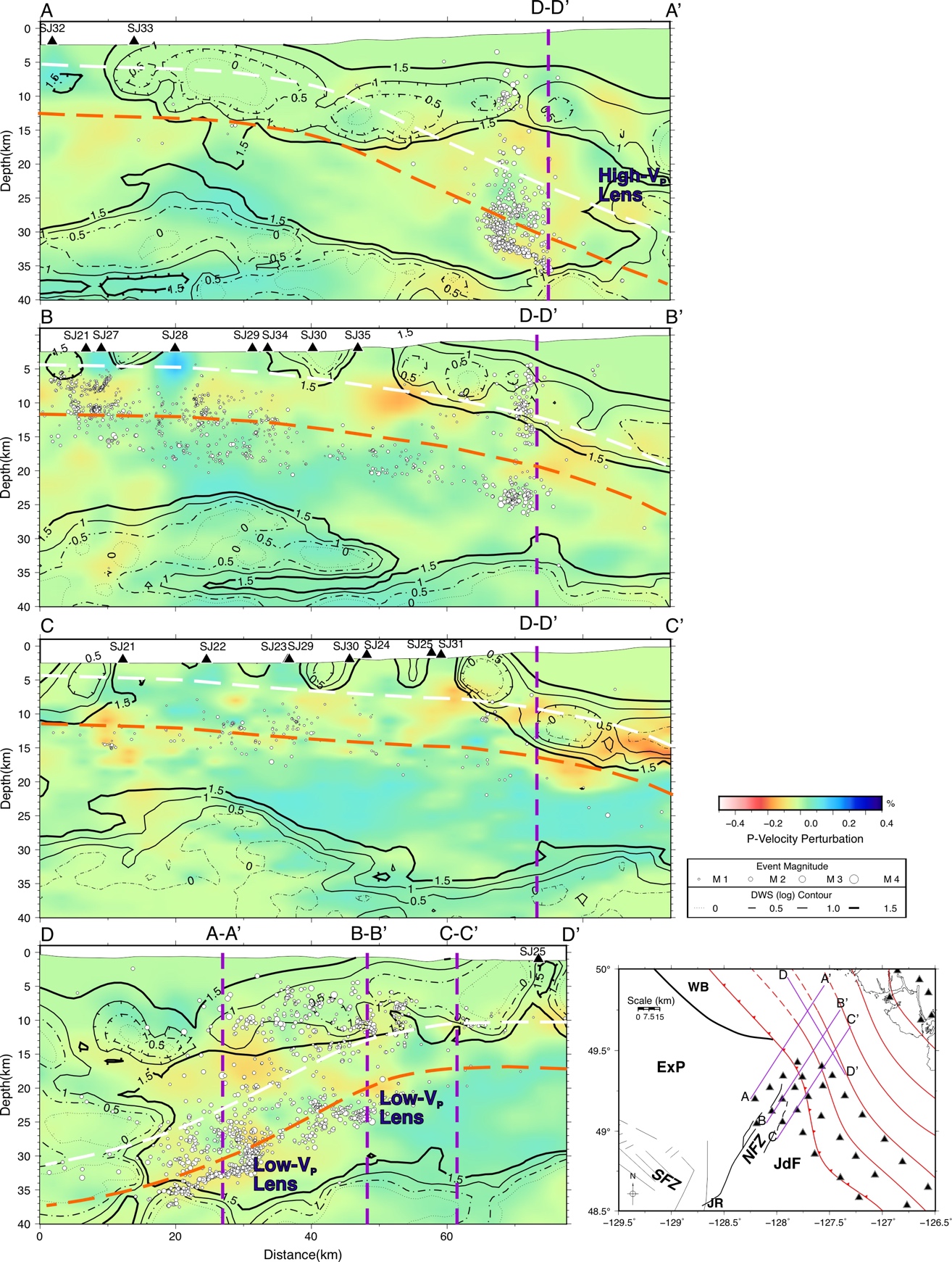


Figure S4. Cross-section profiles of seismic tomography with raypath density DWS contours

. Seismic tomography is presented in the % perturbation of V_P_ from the initial 1-D velocity model (Spence et al., 1985). See the Figure 5 caption for additional information.

Table S1. Station coordinates for the SJ2 network.

| **Station** | FLYN | HSNT | MLSP | MTCH | PLMP | NMSH | S01 | S02 | S03 | S04 | S05 | S06 | S07 | S08 | S09 |
| --- | --- | --- | --- | --- | --- | --- | --- | --- | --- | --- | --- | --- | --- | --- | --- |
| **Latitude (º)** | 49.8268 | 49.7158 | 49.8551 | 49.7885 | 50.0048 | 49.6882 | 48.1922 | 48.3084 | 48.451 | 48.5849 | 48.709 | 48.8575 | 48.9898 | 48.3228 | 48.4011 |
| **Longitude (º)** | -126.93 | -126.5332 | -126.5625 | -126.4631 | -126.7735 | -126.6336 | -126.6113 | -126.8183 | -127.0029 | -127.2212 | -127.3971 | -127.6407 | -127.753 | -126.3638 | -126.576 |
| **Elevation (km)** | 0.047 | 0.068 | 0.228 | 0.005 | 0.111 | 0.104 | -2.49 | -2.557 | -2.57 | -2.563 | -2.546 | -2.538 | -2.521 | -1.172 | -1.554 |

| **Station** | S10 | S11 | S12 | S13 | S14 | S15 | S16 | S17 | S18 | S19 | S20 | S21 | S22 |
| --- | --- | --- | --- | --- | --- | --- | --- | --- | --- | --- | --- | --- | --- |
| **Latitude (º)** | 48.5358 | 48.7262 | 48.8431 | 48.9587 | 49.0929 | 48.4848 | 48.6491 | 48.809 | 48.949 | 49.1008 | 49.2199 | 49.0575 | 49.1313 |
| **Longitude (º)** | -126.7664 | -127.0698 | -127.2512 | -127.4017 | -127.5791 | -126.3695 | -126.5674 | -126.8088 | -126.9788 | -127.189 | -127.3553 | -127.9457 | -127.8082 |
| **Elevation (km)** | -1.42 | -2.045 | -2.057 | -2.066 | -1.976 | -1.016 | -1.137 | -1.341 | -1.069 | -1.603 | -1.411 | -2.502 | -2.504 |

| **Station** | S23 | S24 | S25 | S26 | S27 | S28 | S29 | S30 | S31 | S32 | S33 | S34 | S35 | TAHS | ZBLS |
| --- | --- | --- | --- | --- | --- | --- | --- | --- | --- | --- | --- | --- | --- | --- | --- |
| **Latitude (º)** | 49.2147 | 49.2809 | 49.3452 | 49.0468 | 49.1244 | 49.1989 | 49.2709 | 49.3363 | 49.4025 | 49.2003 | 49.2704 | 49.342 | 49.4268 | 49.9348 | 49.9898 |
| **Longitude (º)** | -127.7025 | -127.5639 | -127.4741 | -128.1916 | -128.0469 | -127.9477 | -127.829 | -127.7584 | -127.5732 | -128.2063 | -128.0662 | -127.9403 | -127.8038 | -126.6484 | -126.8567 |
| **Elevation (km)** | -2.495 | -1.804 | -1.505 | -2.461 | -2.469 | -2.478 | -2.468 | -2.445 | -1.799 | -2.435 | -2.436 | -2.443 | -2.422 | 0.068 | 0.03 |

**Supplemental Table A1.** Arrival information for the hypocentres from SeaJade I and II.

The arrivals are included in the comma-separated file ‘Supplemental_Table_A1.txt’. Associated arrival information is provided in the following format:

origin ID, arrival ID, station of arrival, arrival datetime, arrival phase, channel of arrival

**Supplemental Table A2.** Hypocenter information for the relocated events from SeaJade I and II.

The locations are included in the file ‘Supplemental_Table_A2.xlsx’. The columns are organized as follows:

Origin ID, latitude, longitude, depth, X(m), Y(m), Z(m), EX(m), EY(m), EZ(m), year, month, day, hour, minute, second, magnitude, NCCP, NCCS, NCTP, NCTS, RCC(s), RCT(s), CID.

The X, Y, and Z parameters are measured in meters from the cluster centroid. EX, EY, and EZ are the estimated LSQR errors in E-W, N-S, and depth, respectively. NCCP, NCCS, NCTP, and NCTS, are the numbers of P and S phases used in the cross-correlation and travel-time difference datasets to locate the earthquakes, respectively. RCC and RCT are the time residuals, in seconds, for the cross-correlation and travel-time difference datasets, respectively.

**Supplemental Table B1.** Focal mechanism information for events from SeaJade II.

The focal mechanisms are included in the file ‘Supplemental_Table_B1.xlsx’. Focal mechanisms from the Nootka Sequence have bolded italicized IDs. The columns are organized as follows:

ID, Date, Latitude, Longitude, Depth, Magnitude, Strike, Dip, Rake, FP_unc, Aux_unc, num_P_pol, Wght % Misfit, Rank, Probability, num_SP, and Mode.

The depth parameter is measured in km. The magnitude parameter is measured in local magnitude (M_L_). The parameters FP_unc and Aux_unc are the uncertainties for the primary and auxiliary fault planes, given in degrees. The parameter num_P_pol is the number of *P* first-motion polarities used in calculating the focal mechanism. Wght % Misfit is the weighted percent misfit of first motions. Rank can range from A to F (although only A-ranked data are provided), and is a measure of focal mechanism quality. Probability is a measure of how close the mechanism is to the true solution. num_SP is the number of *S/P* ratios used in calculating the focal mechanism. Mode is the failure mode of the focal mechanism; classification is derived from Alvarez-Gomez (2009): N – normal, R – reverse, S – strike-slip, N-S – normal oblique strike-slip, R-S – reverse oblique strike-slip, S-N – strike-slip oblique normal, S-R, strike-slip oblique reverse.

**Supplemental Table C1.** P-wave tomography model from SeaJade II data.

The model is included in the file ‘Supplemental_Table_C1.txt’, and the columns are organized as follows:

Longitude, latitude, depth, V_P_

**Supplemental Table C2.** S-wave tomography model from SeaJade II data.

The model is included in the file ‘Supplemental_Table_C2.txt’, and the columns are organized as follows:

Longitude, latitude, depth, V_S_
